# CsbZIP1-CsMYB12 mediates the production of bitter-tasting flavonols in tea plants (*Camellia sinensis*) through a coordinated activator–repressor network

**DOI:** 10.1038/s41438-021-00545-8

**Published:** 2021-05-01

**Authors:** Xuecheng Zhao, Xiangsheng Zeng, Ning Lin, Shuwei Yu, Alisdair R. Fernie, Jian Zhao

**Affiliations:** 1 State Key Laboratory of Tea Plant Biology and Utilization, Anhui Agricultural University, 230036 Hefei, China; 2 College of Agronomy, Anhui Agricultural University, 230036 Hefei, China; 3 Max-Planck-Institute of Molecular Plant Physiology, Am Mühlenberg 1, 14476 Potsdam-Golm, Germany

**Keywords:** Light responses, Secondary metabolism

## Abstract

Under high light conditions or UV radiation, tea plant leaves produce more flavonols, which contribute to the bitter taste of tea; however, neither the flavonol biosynthesis pathways nor the regulation of their production are well understood. Intriguingly, tea leaf flavonols are enhanced by UV-B but reduced by shading treatment. *CsFLS*, *CsUGT78A14, CsMYB12*, and *CsbZIP1* were upregulated by UV-B radiation and downregulated by shading. CsMYB12 and CsbZIP1 bound to the promoters of *CsFLS* and *CsUGT78A14*, respectively, and activated their expression individually. CsbZIP1 positively regulated *CsMYB12* and interacted with CsMYB12, which specifically activated flavonol biosynthesis. Meanwhile, *CsPIF3* and two MYB repressor genes, *CsMYB4* and *CsMYB7*, displayed expression patterns opposite to that of *CsMYB12*. CsMYB4 and CsMYB7 bound to *CsFLS* and *CsUGT78A14* and repressed their CsMYB12-activated expression. While CsbZIP1 and CsMYB12 regulated neither *CsMYB4* nor *CsMYB7*, CsMYB12 interacted with CsbZIP1, CsMYB4, and CsMYB7, but CsbZIP1 did not physically interact with CsMYB4 or CsMYB7. Finally, CsPIF3 bound to and activated *CsMYB7* under shading to repress flavonol biosynthesis. These combined results suggest that UV activation and shading repression of flavonol biosynthesis in tea leaves are coordinated through a complex network involving *CsbZIP1* and *CsPIF3* as positive MYB activators and negative MYB repressors, respectively. The study thus provides insight into the regulatory mechanism underlying the production of bitter-tasting flavonols in tea plants.

## Introduction

Tea plants (*Camellia sinensis*) synthesize diverse flavonoids, such as catechins, flavonols, and anthocyanins and their derivatives, at significant levels in tender tissues, such as apical buds and young leaves. These flavonoids, together with caffeine and theanine, constitute the major bioactive secondary metabolites in teas, contributing to their pleasant flavors, rich tastes, and multiple health benefits, features that are of vast importance given that tea is the most consumed nonalcohol beverage in the world^1–3^. Both catechins, primarily epigallocatechin-3-gallate (EGCG), and flavonols, mainly kaempferol glycosides, are the major contributing factors to the bitter and astringent tastes, with very low sensory doses being recognized by the human tongue^4,5^. The tender shoot tips of tea plants are usually picked during early spring, when the weather is still cool and misty with less sunlight radiation, to ensure the highest quality of teas. It has been well documented that these tender shoot tips contain higher levels of amino acids, mainly theanine, and fewer bitter-tasting catechins and flavonols in spring^6^. Indeed, tea plant leaves often accumulate higher levels of flavonols and catechins, which may result from high-intensity light irradiation during the summer-autumn seasons^7^. Light intensity and light quality significantly affect the accumulation of characteristic secondary metabolites in tea plant leaves^8,9^. Both red and blue light promote the production of catechins and caffeine^9^, while UV-A and UV-B promote the production of anthocyanins^10^. Thus, shading of tea plants has been frequently applied in tea gardens to reduce the contents of these bitter-tasting and astringent flavonoids in tea plant leaves^8,11,12^. Transcriptome and metabolite profiling revealed that transcription factors (TFs) involved in light perception and signaling may be connected with TFs regulating flavonoid biosynthetic genes^8,9^. However, the genetic factors and detailed molecular mechanisms underlying how light exposure promotes and shading reduces the accumulation of tea flavonoid contents in tea plant leaves are not yet understood^8,13,14^. Since the levels of flavonols significantly affect tea flavor and health function, it is highly desirable to understand how environmental factors regulate their biosynthesis.

Flavonols are a particular class of flavonoids that are present in most green leaves. The biosynthesis and regulation of flavonol glycosides in tea plant leaves attracted our attention, as they are the major bitter-tasting substances in tea leaves grown under strong light conditions. The branched flavonol pathway has been studied extensively, including work on common shared enzymes such as F3H, F3’H, F3′5′H, as well as specific enzymes such as flavonol synthase (FLS) and UDP-glucose: flavonol glycosyltransferases (UGTs)^6,15^. Flavonol-specific FLS competes for the precursor dihydroflavonols with dihydroflavonol 4-reductase (DFR), leading to varying amounts of anthocyanin and proanthocyanidin synthesis^16,17^. Flavonol synthesis is also highly regulated at the transcriptional level by several tissue-specifically expressed R2R3-MYB transcriptional activators, such as Arabidopsis *AtMYB11*, 12, and 111^18^, apple *MYB12* and *MYB22*^19^, and grapevine *VvMYBF1*^20^. Meanwhile, R2R3-MYB repressors, such as Arabidopsis *AtMYB7* and *AtMYB4*, have been demonstrated to be regulators of flavonoid biosynthesis in plants^21^. These activators and repressors, as well as other TFs, are specifically responsive to certain environmental cues and together form a regulatory network to fine tune flavonol biosynthesis in plants^22,23^.

Light is a crucial signal that affects plant growth and development and involves light receptor phytohormones, signaling proteins, and many downstream effectors, including metabolic enzymes and developmental regulators^24,25^. Several photoreceptors are characterized to respond to different wavelengths of light: the red/far-red light photoreceptor phytochromes, the blue/UB-A light photoreceptor cryptochromes and phototropins, and the UV-B light photoreceptor UVR8^26,27^. These activated photoreceptors directly or indirectly modify the stability of primary TFs such as ELONGATED HYPOCOTYL 5 (HY5), PHYTOCHROME INTERACTING FACTOR 3 (PIF3), and PHYTOCHROME INTERACTING FACTOR 4 (PIF4)^28^. It is well known that plants accumulate increased levels of flavonols under high light conditions or UV-B irradiation than under regular light irradiation^29^. The E3 ubiquitin ligase CONSTITUTIVE PHOTOMORPHOGENIC1 (COP1) negatively controls photomorphogenesis by interacting with SUPPRESSOR OF PHYTOCHROME A (SPA1–SPA4) proteins to inhibit photomorphogenic growth^30^. HY5 is a key photomorphogenesis-promoting factor downstream of COP1 and is destabilized by COP1 in darkness^31^. HY5 directly regulates the promoters of thousands of genes involved in plant development and flavonoid biosynthesis^32^. HY5 can regulate flavonol biosynthesis by mediating UV-B or light irradiation-induced AtMYB12 activation and flavonol accumulation^33^. In the second branch of the pathway, a basic helix-loop-helix (bHLH) TF and PHYTOCHROME-INTERACTING FACTORs (PIFs) promote skotomorphogenesis and repress photomorphogenesis under red and far-red light conditions^34^. PIFs play diverse roles in plant growth and development by positively or negatively regulating a large number of downstream genes^35^. PIF3 plays multiple roles in light signaling as a negative factor in hypocotyl elongation and anthocyanin biosynthesis and a positive factor in the plant shading response^36^. In contrast to the case for HY5, light irradiation leads to PIF3 protein phosphorylation and degradation^37^. HY5 and PIFs are oppositely regulated by light. PIF3 and HY5 interact with cryptochromes and UVR8 to regulate light-responsive genes^38^. HY5 and PIF1/PIF3 interact with each other directly and antagonistically regulate reactive oxygen species-responsive genes and the greening of etiolated seedlings upon light irradiation^39^. However, how these factors are involved in light- or shading-regulated flavonoid biosynthesis remains unknown. An improved understanding of these mechanisms in tea plants is highly important given the common application of shading to tea plants to mediate the quality of tea production^8,9,11^.

This study attempts to dissect the comprehensive regulatory network mediating light- and shading-regulated biosynthesis of flavonols in tea plants. UV-B or shading treatment prominently altered bitter-tasting flavonol contents in tea plant leaves. UV-B radiation acted through *CsbZIP1-CsMYB12* on the key flavonol biosynthetic genes *CsFLS* and *CsUGT78A14*, while shading repressed flavonol biosynthesis not only by inactivation of HY5-like *CsbZIP1* but also via activation of *CsPIF3*, which further activated the MYB repressor genes *CsMYB4* and *CsMYB7*. Transactivation assays revealed that CsMYB4 and CsMYB7 repressed *CsFLS*. We therefore demonstrated a complex regulatory network composed of both activators and repressors in the regulation of bitter-tasting flavonol production by UV-B exposure and shading treatment in tea plant leaves.

## Materials and methods

### Plant material and growth conditions

“Shu Cha Zao”, “Long Jing”, “Huang Shan Bai Cha”, “Zi Juan”, and “Huang Kui” tea plants were grown in the experimental tea garden of Anhui Agricultural University, Anhui, China (31°. 55′ North, 117°. 12′ East; Hefei City, Anhui Province, China). UV-B conditions (300 μW cm^−2^, photoperiod of 12 h per day) were provided using a special lamp (PHILIPS NARROWBAND TL 20 W, Poland) with a characteristic peak at 311 nm, 25/18 °C light/dark. The shading experiment consisted of two treatments: tea plants with natural growth (control) and tea plants with 90% shading treatment. The nets were placed over the plants on 27 July 2019, when a new round of bud burst started. The second leaves of tea of the same growth stage were collected throughout shading treatments (0 h, 4 h, 8 h, 12 h, 2 days, 4 days, 8 days, and 14 days after shading). All samples were stored at −80 °C until use. Methyl jasmonate (MeJA) treatment experiments were performed as described previously^40^. Tea plant leaves sprayed with 100 µM MeJA solution or distilled water (control) were collected at 0, 12, 24, and 48 h after the onset of treatment. The polyethylene glycol (PEG) and NaCl treatment experiments were performed as described previously^41^. Briefly, tea plant seedlings were treated with 25% PEG or 200 mM NaCl for 0, 24, 48, and 72 h to mimic drought and salinity stress conditions, respectively. For the cold treatment experiments, tea plant leaves were collected during the cold acclimation (CA) process. Control (CK): 25 °C; CA_1_-6 h: 10 °C for 6 h; CA_1_-7 days: from 10 °C to 4 °C for 7 days; CA_2_-7 days: from 4 °C to 0 °C for 7 days; DA-7d: recover under 25 °C to 20 °C for 7 days, as described previously^42^. Transcriptome data from experiments with tea cv. “Shu Cha Zao” were retrieved from the tea plant information archive (http://tpia.teaplant.org/index.html).

### Chemical standards and other chemicals

All flavonoid standards were of analytical grade, including myricetin 3-*O*-galactoside (M-3-O-Gal), myricetin 3-*O*-glucoside (M-3-*O*-Glu), quercetin-3-*O*-glucoside (Q-3-*O*-Glu), quercetin-3-*O*-rutinoside (Q-3-*O*-R), quercetin-3-O-galactopyranoside (Q-3-O-Gal), kaempferol 3-*O*-galactosylrutinoside (K-3-*O*-Galact), kaempferol-3-*O*-glucoside (K-3-*O*-Glu), kaempferol-3-O-galactoside (K-3-*O*-Gal), and kaempferol-7-*O*-glucoside (K-7-*O*-Glu), which were purchased from Sigma Chemicals Co. (Sigma-Aldrich, USA). Methanol, acetonitrile, and acetic acid of chromatographic grade were purchased from Shanghai GuoMei Pharmaceutical Co. UPLC-grade water was prepared from distilled water using a Milli-Q system (Millipore Laboratory, Bedford, MA, USA).

Flavonols were detected by ultrahigh-performance liquid chromatography (UPLC) on an Agilent InfinityLab Poroshell HPH-C18 instrument (4.6 × 100 mm, 2.7 μm, Agilent, Santa Clara, CA, USA). The samples (5 μL injection volume) were loaded on an Inertsil ODS-3 column and eluted at a flow rate of 1.0 mL/min. Mobile phases A and B were composed of 0.1% acetic acid in distilled water and acetonitrile, respectively. The elution program was as follows: calibration with 95% A (1% acetic acid) and 5% B (100% acetonitrile), a linear gradient from 5 to 10% B for 0 − 2 min, from 10 to 20% B for 2−15 min, from 20 to 30% B for 15−30 min, and from 30 to 55% B for 30 − 55 min was performed, followed by washing and equilibration. The flavonols were detected at a wavelength of 350 nm, and the column temperature was set at 35 °C^6^.

### Detection of flavonols from leaves

Leaves of “Shu Cha Zao”, “Long Jing”, “Huang Shan Bai Cha”, “Zi Juan”, and “Huang Kui” tea plants and different tissues of “Shu Cha Zao” were ground to a fine powder using a mortar and pestle in liquid nitrogen. The powered leaf samples (0.2 g) were extracted with 2 mL 80% methanol by sonication at room temperature for 5 min, followed by centrifugation at 4500×*g* for 10 min. The residues were re-extracted twice by this method. The supernatants were filtered through a 0.22-μm membrane. Flavonols were analyzed according to previously described UPLC methods^6^.

### Quantitative real-time PCR

Total RNA was isolated from leaves with RNAiso Plus and RNAiso Mate for Plant Tissue Kits (TaKaRa, China). Double-stranded cDNA was prepared using the Super SMART PCR cDNA Synthesis Kit (Clontech, Palo Alto, USA) following the manufacturer’s instructions. Quantitative real-time PCR (qRT-PCR) was carried out using the SYBR Green method for the detection of double-stranded PCR products (TaKaRa, Dalian, China). An IQ5 real-time PCR detection system (Bio-Rad) was utilized in this study as previously described. The tea β-actin gene was used as an internal reference gene (HQ420251.1, https://www.ncbi.nlm.nih.gov/nuccore/). qRT-PCR data were generated using an Applied Biosystems 7900HT instrument, and analyses were performed using SDS software (Applied Biosystems). PCR efficiencies were calculated using LinReg software. The primers for representative genes in this study were designed by Primer Premier 5.0 software (PREMIER Biosoft company; Tables S1 and S2).

### Sequence alignment and phylogenetic analysis

In this study, amino acid sequence alignment analysis of MYBs was conducted using DNAMAN 8.0 software (Lynnon, Quebec, Canada). A phylogenetic analysis using the amino acid sequences of MYB members was performed using MEGA 7.0 software (http://www.megasoftware.net/, Mega Software, State College, PA, U.S.A.), and a phylogenetic tree was constructed using neighbor-joining distance analysis. The tree nodes were evaluated with the bootstrap method for 1000 replicates, and the evolutionary distances were computed using the p-distance method. Sequence information used in the phylogenetic tree is shown in Table S3.

### Subcellular localization

Sequence information on *CsMYB4*, *CsMYB7*, *CsMYB12* and *CsPIF3* was obtained from the tea plant genome (http://tpia.teaplant.org/). Sequence information of *CsbZIP1* was obtained from transcriptome data^8^. The ORFs of *CsMYB4*, *CsMYB7*, *CsMYB12*, *CsbZIP1*, and *CsPIF3* within the entry vector pDONR211 were cloned into the destination binary vector, namely, PK7WGF2.0, for subcellular localization studies. Positive vectors in which the ORF was fused at the N-terminus of GFP were obtained and named PK7WGF2.0-CsMYB4, PK7WGF2.0-CsMYB7, PK7WGF2.0-CsMYB12, PK7WGF2.0-CsbZIP1, and PK7WGF2.0-CsPIF3, respectively. As described above, the plasmids were introduced into *A. tumefaciens* strain GV3101 to select a positive colony for infiltration of *Nicotiana benthamiana*. After 48 h of infiltration, leaves were examined using an Olympus FV1000 confocal microscope (Olympus, Tokyo, Japan). GFP fluorescence signals were excited with a 488-nm laser, and the emitted light was recorded from 500 to 530 nm to display the subcellular localization of CsMYB4, CsMYB7, CsMYB12, CsbZIP1, and CsPIF3.

### Overexpression of *CsMYB12* in soybean hairy roots

*CsMYB12* was constructed in pB2WG7 for overexpression and GUS as a control. These confirmed constructs were transformed into *Agrobacterium rhizogenes* strain K599 by electroporation. Positive colonies were selected on LB-agar medium containing selective antibiotics at 28 °C. Positive K599 colonies were used to generate hairy roots from germinated soybean (*Glycine max*) seeds. Soybean cultivar “Tianlong #1” seeds were surface sterilized and germinated in Petri dishes containing sterilized filter paper. The surfaces of 7-day-old green cotyledons were wounded and infected with K599 harboring the vectors for overexpression. The transgenic hairy roots were subjected to semiquantitative or qRT-PCR analyses to validate their identity. The transgenic hairy roots were maintained on half-strength Murashige and Skoog medium (MS medium) containing 7.5 mg L^−1^ phosphinothricin (ppt) for selection in a growth chamber at 23 °C with a 16 h/8 h light/dark photoperiod and subculture every 3–4 weeks.

### Yeast one-hybrid and two-hybrid assays

Yeast one-hybrid (Y1H) and two-hybrid assays were conducted as previously described^43^. Yeast one-hybridization assays were performed using the Matchmaker Gold Yeast One-Hybrid System (Clontech). To construct transcription factor-expressing cassettes, the ORFs of *CsMYB4*, *CsMYB7*, *CsMYB12*, *CsPIF3*, and *CsbZIP1* were recombined into the pGADT7 vector (Clontech, Palo Alto, USA). The cloned promoter fragments of *CsMYB12*, *CsMYB7*, *CsFLS*, and *CsUGT78A14* were inserted into the pHIS2.1 vector. The yeast strain Y187 containing the recombinant pHIS2.1 vector was grown on –Trp–Leu (–T–L) screening medium for 3 days at 30 °C. Then, the interactions between the MYB TFs and promoter fragments were detected on medium lacking Trp, Leu and His (–T–L–H) for 3–5 days at 30 °C. Empty pGADT7 vectors were used as controls.

For yeast two-hybrid assays (Y2H), the ORFs of the *CsMYB4, CsMYB7, CsbZIP1*, and *CsMYB12* genes were recombined into the pGBKT7 and pGADT7 vectors, respectively (Clontech, Palo Alto, USA). The recombinant plasmids were cotransformed into the yeast strain AH109 and cultured on medium lacking Trp and Leu (–T–L) for 3 days at 30 °C. For interaction screening, the yeast cells were transferred to medium lacking Trp, Leu, His and adenine (–T –L–H–A) with X-gal for 3–5 days at 30 °C. Empty pGADT7 and pGBKT7 vectors were used as controls.

### Luciferase reporter assay

The ORFs of *CsMYB4*, *CsMYB7*, *CsMYB12*, *CsbZIP1*, and *CsPIF3* were recombined into the P2GW7 effector expression system, as described previously^43^. The cloned promoter fragments of *CsMYB12*, *CsMYB7*, and *CsFLS* were inserted into the pGreen-0800-LUC reporter. Protoplasts derived from *Arabidopsis thaliana* were used as the materials for transient transfection. Each transfection contained the GUS plasmid for normalization. For transient transfection, 1 μL of GUS plasmid, 5 μL of LUC reporter, and 10 μL of effector were mixed together and transformed into *Arabidopsis thaliana* protoplasts using 40% polyethylene glycol. After reaction at 24 °C for 12 h, the LUC and GUS activities were tested using a Multimode Plate Reader (Victor X4, PerkinElmer, http://www.perkinelmer.com/). The promoter activity was calculated by the ratio of LUC to GUS activity.

### Suppression of *CsMYB12* and *CsbZIP1* in tea shoot tips by using candidate antisense oligonucleotides

Since tea plant transformation technology has not yet been developed, knockdown of the target gene with antisense oligonucleotides (asODN) containing the segment complementary to the target gene was used to examine how *CsbZIP1* and *CsMYB12* affect flavonol synthesis in tea shoot tips^44,45^. The antisense oligonucleotides (asODN) were selected by using Soligo software (http://sfold.wadsworth.org/cgi-bin/soligo.pl) with *CsMYB12* and *CsbZIP1* as input sequences (Table S2). To silence the genes, fresh shoot tips (with the apical bud and 1st leaf) of the tea plant variety “Shu Cha Zao” were incubated in 2 ml Eppendorf tubes containing 40 μM asODN-*CsMYB12* or asODN-*CsbZIP1* solution for various times. Shoot tips incubated with the same concentrations of sense oligonucleotides (sODN) were used as the control. Shoot tips were sampled at different time intervals for RNA and flavonol analysis.

### Bioinformatic analysis

The GenBank accession numbers for genes characterized in the study were as follows: *CsMYB12* (MT498592), *CsMYB4* (MT498593), *CsMYB7* (MT498594), *CsbZIP1* (MT498595), and *CsPIF3* (MT498596). A multiple sequence alignment of the amino acid sequences of the CsMYB TF proteins of tea plant, rice and Arabidopsis was generated with ClustalW. An unrooted phylogenetic tree based on the sequence alignments was constructed using MEGA 7.0 software (http://www.megasoftware.net/) and the neighbor-joining method with the following parameters: pairwise alignment and 1000 bootstrap replicates. All resulting heatmaps of expression were structured by the pheatmap R package.

### Statistical analysis

All experimental data are taken from at least three independent experiments. For *C. sinensis* shoot tip antisense inhibition experiments, at least 10 independent plants were analyzed with three repeats each. For Y2H assays, subcellular localization, and transgenic hairy root experiments, representative pictures are shown. Differences at the 95% confidence level in two-tailed Student’s *t* test were considered significant.

## Results

### UV-B and shading treatments regulated flavonol biosynthesis in tea plant leaves

**Fig. 1:**
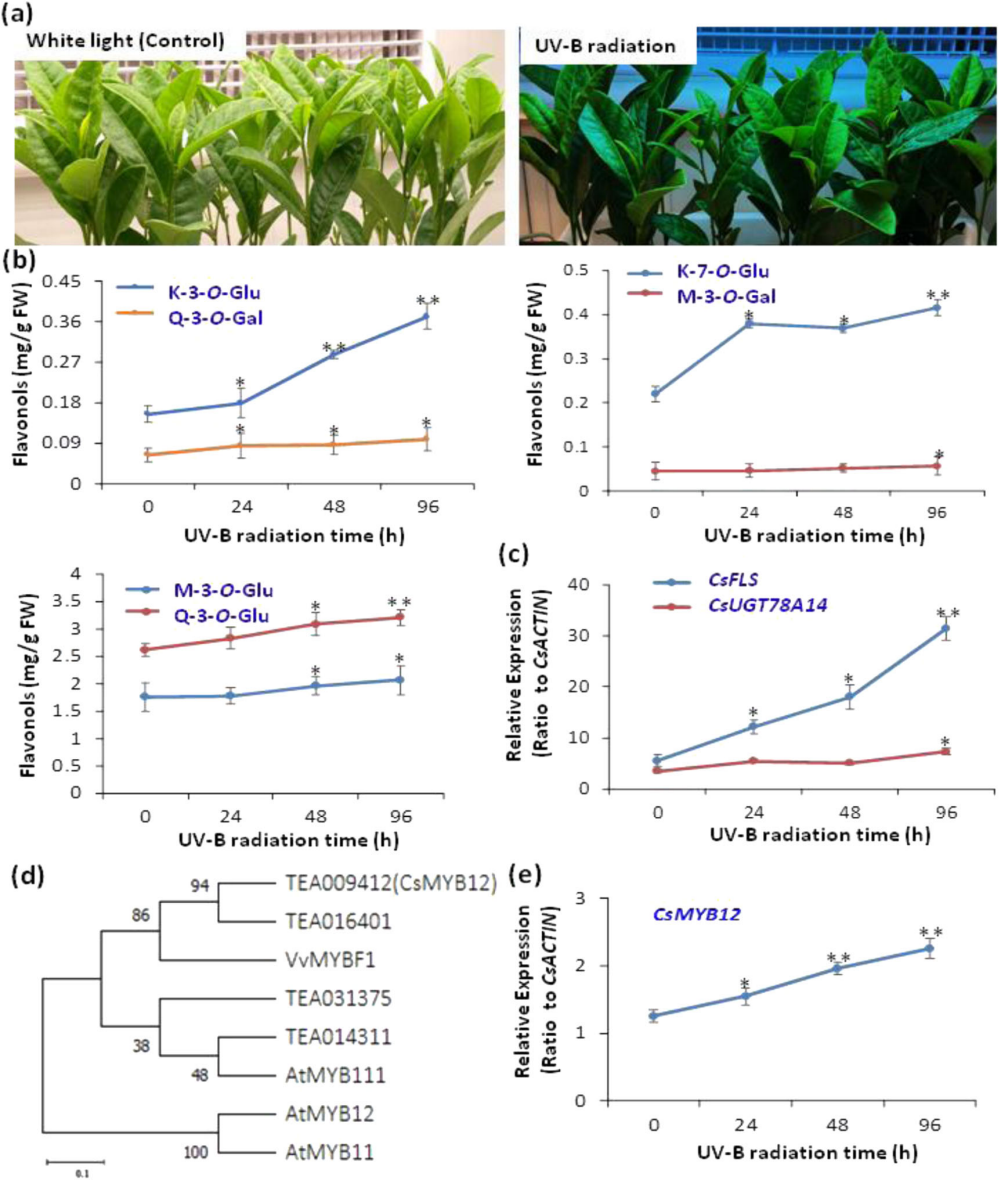
Effects ofg on flavonol biosynthesis and gene expression in tea plant leaves. **a** UV-B radiation of tea plant seedlings. UV-B conditions (300 μW cm^−2^, photoperiod of 12 h per day, provided with a special lamp (PHILIPS NARROWBAND TL 20 W, Poland) with a characteristic peak at 311 nm. at 25/18 °C (light/dark) with those under regular white light as controls. **b** Content changes in M-3-*O*-Gal, M-3-*O*-Glu, Q-3-*O*-Glu, Q-3-*O*-Gal, K-7-*O*-G, and K-3-*O*-G in tea plant leaves under UV-B radiation in comparison with control (under regular white light). **c** Expression levels of *CsFLS* and *CsUGT78A14* as a function of UV-B radiation compared with the control. **d** Phylogenetic identification of CsMYB12 and R2R3 MYB homologs from other plants. *Vitis vinifera* MYBF1: VvMYBF1; *Arabidopsis thaliana* MYB111, 12, 11: AtMYB111, AtMYB12, AtMYB11. **e** Expression patterns of *CsMYB12* in tea plant leaves under UV-B treatment compared with controls. Differences between UV-B irradiated and untreated (control) samples were analyzed. Data were from three independent experiments and expressed as the means ± SD. (*n* = 3). The differences were analyzed in two-tailed comparisons with the control, and *p < 0.05; **p < 0.01 in Student’s *t* test

**Fig. 2:**
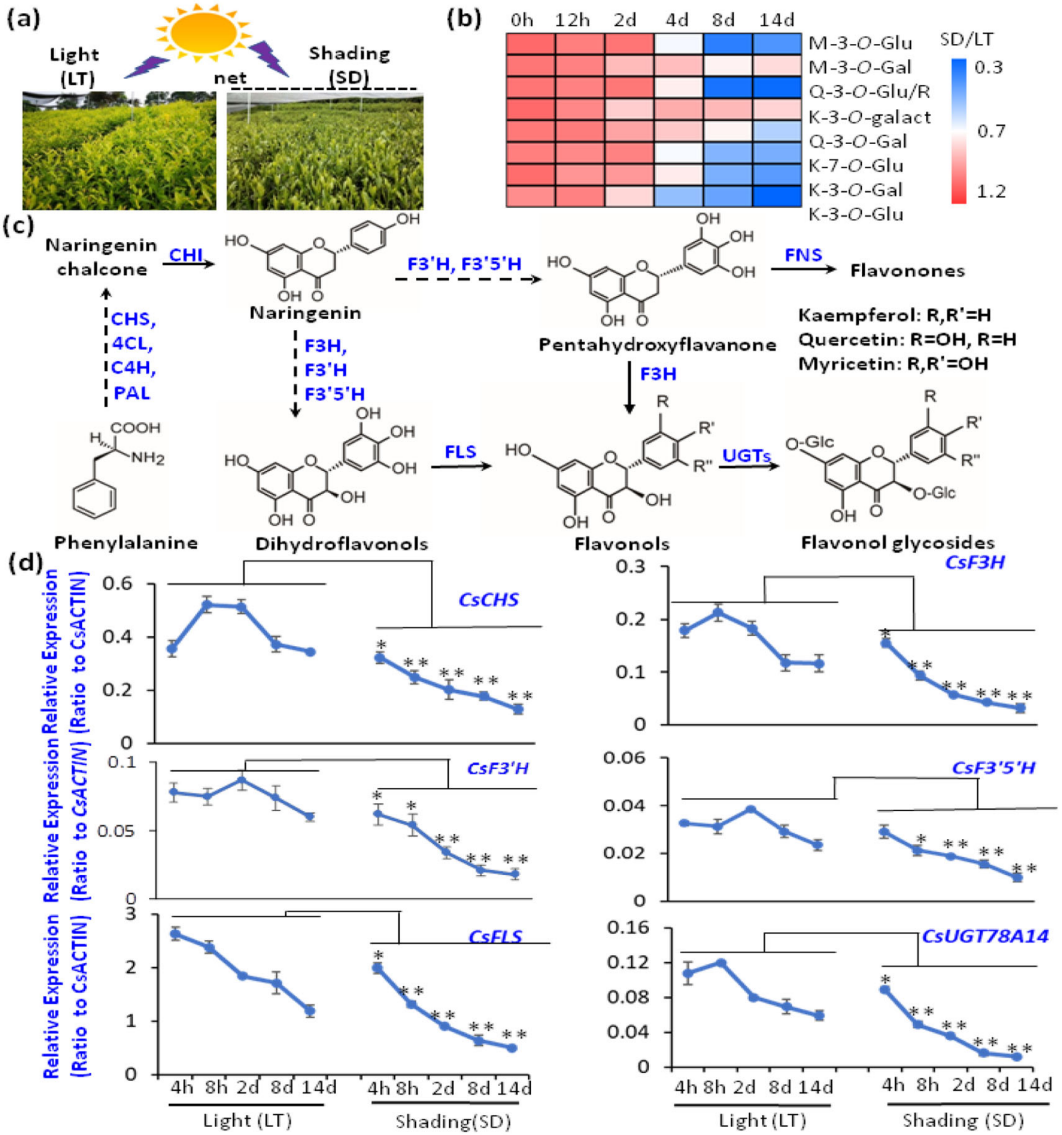
Effects of shading treatment on flavonol biosynthesis and gene expression in tea plant leaves. **a** Tea plants grown in tea gardens treated with shading (SD, 90% blocked light) in comparison with plants grown under regular light radiation (LT). **b** Changes in major flavonols in tea plant leaves under shading treatment. Data are expressed as the ratio of SD:LT (shading:light). **c** Key genes involved in the flavonoid pathway for the biosynthesis of flavonols in tea plants. **d** Effects of shading on metabolic gene expression in tea plant leaves. Differences between SD and LT (control) were analyzed. Data were from three independent experiments and expressed as the means ± S.D. (*n* = 3). Differences were analyzed in two-tailed comparisons of treatment and control, *p < 0.05; **p < 0.01 in Student’s *t* test

To understand the molecular regulatory mechanisms underlying the regulation of flavonol synthesis by light exposure, we conducted both UV-B radiation and shading treatment experiments on tea plant seedlings hydroponically grown in SK nutrient solution and on tea plants grown in tea gardens. In tea plant seedlings grown hydroponically under UV-B conditions (300 μW cm^−2^, photoperiod of 12 h per day, provided with a special lamp (PHILIPS NARROWBAND TL 20 W, Poland; Fig. 1a and S1), M-3*-O-*Gal, M-3*-O-*Glu, Q-3*-O-*Glu, Q-3*-O-*Gal, K-3-*O*-Glu, and K-7*-O-*Glu were increased by 1.5- and 2-fold compared to the control (Fig. 1b). In addition, when we shaded the tea plants grown in tea gardens under sunlight with a thick cloth (Figs. 2a and S2), which allowed only 10% of sunlight to pass through, the flavonol contents in young leaves of the tea plants decreased. Metabolite profiling revealed that light and shading treatments significantly affected flavonoid accumulation in tea leaves. Levels of kaempferol, quercetin, and myricetin glycosides, such as M-3*-O-*Gal, M-3*-O-*Glu, Q-3*-O-*Glu, Q-3*-O-*R, Q-3*-O-*Gal, K-3*-O-*Gal, K-3*-O-*Glu, K-3*-O-*Gal, and K-7*-O-*Glu, were reduced in plants that had received shading compared with controls (Figs. 2b and S3). Several key enzymes are involved in the biosynthesis of flavonols in tea plants, including flavanone 3-hydroxylase (*CsF3H*, TEA023790.1), flavonoid 3′-hydroxylase (*CsF3*′*H*, TEA006847.1), flavonoid 3′,5′-hydroxylase (*CsF3'5*′*H*, TEA026294.1), chalcone synthase (*CsCHS*, TEA023331.1), flavonol synthase (*CsFLS*, TEA006643.1), and UDP-glycose: flavonoid 3-O-glycosyltransferase (*CsUGT*, TEA007509.1; Fig. 2c). The expression patterns of genes associated with flavonol biosynthesis were next analyzed to understand how light regulates flavonol accumulation in tea plant leaves. Two structural genes and a MYB TF (Fig. 1d) of the flavonol biosynthetic pathway, namely, *CsFLS*, Cs*UGT78A14*, and *CsMYB12*, were revealed by qRT-PCR analysis to be significantly activated by UV-B radiation (Fig. 1c, e). In addition, flavonol biosynthetic pathway genes displayed significantly lower expression levels in shaded leaves than in tea plant leaves fully exposed to sunlight (Fig. 2d). Moreover, *CsCOP1* transcript levels were upregulated by shading treatment, but *CsUVR8* transcript levels were repressed (Fig. S4a, b).

### *CsMYB12* mediated light-induced flavonol biosynthesis

**Fig. 3:**
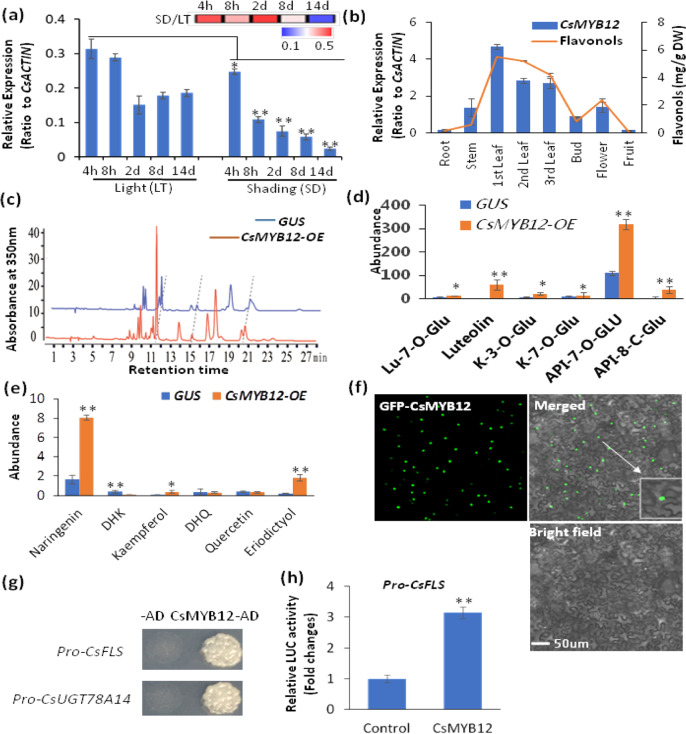
CsMYB12 mediated light-induced flavonol biosynthesis. **a** Effects of shading (SD) on *CsMYB12* expression in comparison with light (LT). **b** Expression patterns of *CsMYB12* in different stages and total flavonoid contents (sums of the contents at different stages). **c** UPLC chromatograms of flavonoid profiles in *CsMYB12*-overexpressing hairy root lines. **d**, **e** Flavonoid compounds in *CsMYB12*-overexpressing hairy root lines and GUS (control). **f** Nuclear localization of the CsMYB12-GFP fusion protein in leaf epidermal cells of *Nicotiana benthamiana*. Bar = 50 µm. **g** Interaction of CsMYB12 with the promoters of *CsFLS* and *CsUGT78A14*. **h** Effects of CsMYB12 on the promoter activity of *CsFLS* with the luciferase reporter assay. Differences between SD and LT (control) were analyzed. Data were from three independent experiments and expressed as the mean ± S.D. (*n* = 3). Differences were analyzed in two tailed with the control, *p < 0.05; **p < 0.01 in Student’s *t* test

We next identified the TFs that may regulate the light-induced or shading treatment-repressed biosynthesis of flavonol. When analyzing transcriptome data from previous experiments, we identified an AtMYB12 homolog MYB TF TEA009412 (tentatively named *CsMYB12*), which is more highly expressed in tea plant varieties Longjing (LJ) and Shu Cha Zao (SCZ) than in Huang Shan Bai Cha (HSBC), Huang Kui (HK), and Zi Juan (ZJ), corresponding to the higher flavonol contents in LJ and SCZ than in HSBC, HK, and ZJ varieties (Fig. S5a, b). Another AtMYB12 homolog, TEA016401, was expressed at very low levels in most tissues and did not respond to light radiation or shading treatment (Figs. S6, S7). Light and shading treatment experiments with SCZ and qRT-PCR verification of *CsMYB12* transcripts also showed that CsMYB12 was repressed by shading treatment, coincident with the reduced total flavonols (Fig. 3a), and that *CsMYB12* transcript levels in various tissues of tea plants were tightly associated with the total flavonol contents in these tissues (Fig. 3b). When *CsMYB12* was overexpressed in soybean hairy roots (Fig. S8), it also triggered significant increases in flavonol and flavonone biosynthesis (Figs. 3c, S9). Metabolite profiling revealed that K-3*-O-*Glu, K-7*-O-*Glu, A-7*-O-*Glu, and A-8-C*-O-*Glu levels were significantly higher in the *CsMYB12*-overexpression (OE) hairy root lines than in the GUS control lines (Fig. 3d). Naringenin, kaempferol, and eriodictyol contents were markedly increased in *CsMYB12-OE* hairy root lines compared with the *GUS* lines (Fig. 3e). Thus, CsMYB12 is a flavonol biosynthesis regulator in tea plants. Consistent with this, GFP-CsMYB12 fusion protein signals in tobacco epidermal cells were observed in the nucleus, suggesting its function as a TF (Fig. 3f). We further investigated the regulatory function of CsMYB12. Yeast one hybrid (Y1H) studies showed that as a nuclear R2R3-MYB TF, CsMYB12 could bind to the promoters of the critical flavonol synthetic genes *CsFLS* and *CsUGT78A14*, whose promoter regions contain several putative MYB-binding cis-elements (Fig. 3g). Transactivation assays using a dual luciferase reporter system showed that *CsMYB12* resulted in 3-fold activation of *CsFLS* (Fig. 3h).

### The light signaling bZIP TF CsbZIP1 regulates *CsMYB12* and *CsFLS*

**Fig. 4:**
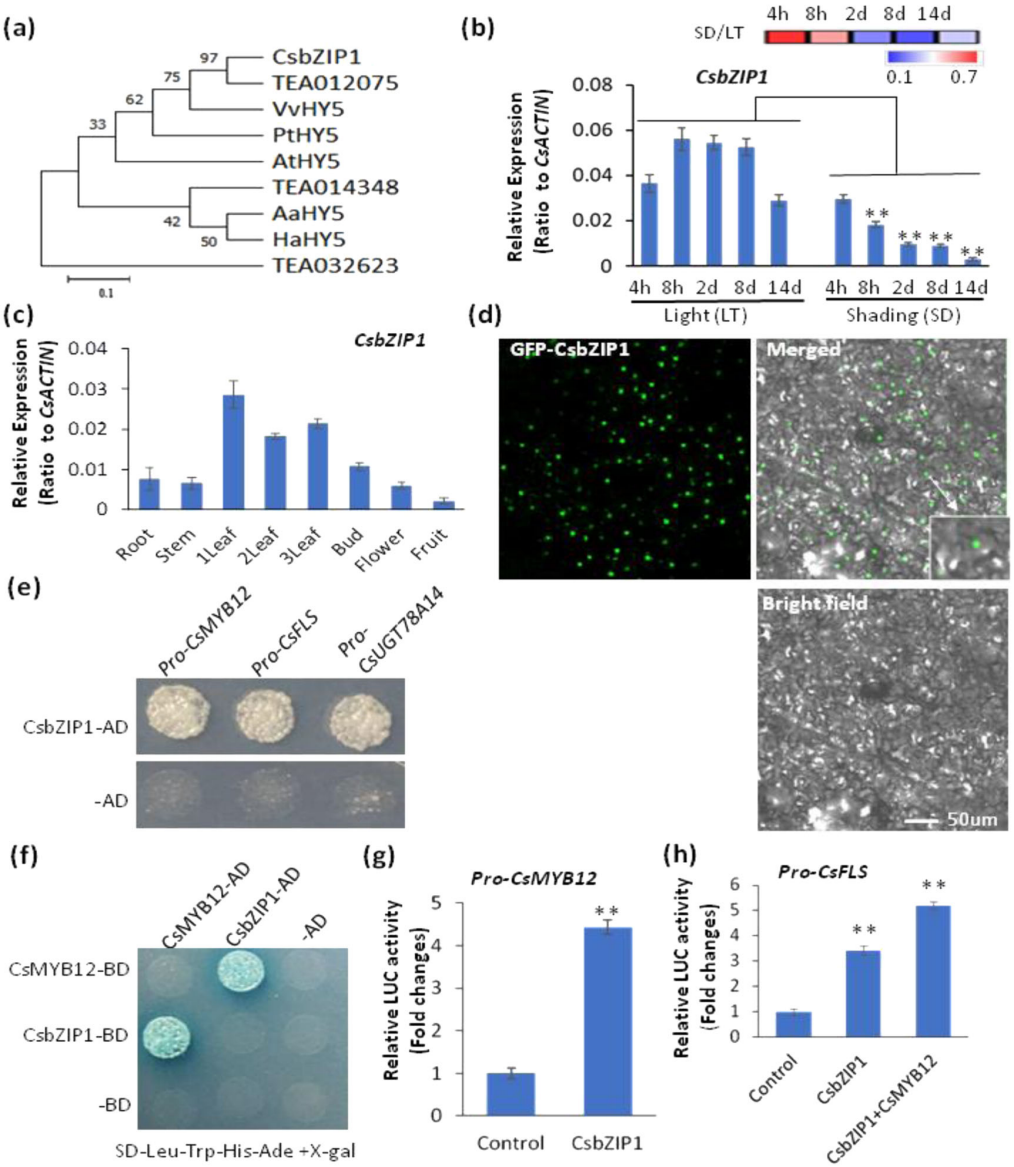
CsbZIP1 regulated flavonol pathway genes in tea plants. **a** Phylogenetic analysis of CsbZIP1 and other bZIP TFs from different plants. *Vitis vinifera* HY5: VvHY5; *Populus trichocarpa* HY5: PtHY5; *Arabidopsis thaliana* HY5: AtHY5; *Artemisia annua HY5:* AaHY5; *Helianthus annuus* HY5: HaHY5. **b** Effects of shading (SD) compared with regular light (LT) on *CsbZIP1* expression in tea plant leaves. **c** Expression profiles of *CsbZIP1* in different tea plant tissues. **d** Nuclear localization of GFP-CsbZIP1 fusion protein in tobacco leaf epidermal cells. Bar = 50 µm. **e** Binding of CsbZIP1 to the *CsMYB12*, *CsFLS* and *CsUGT78A14* promoters in a Y1H assay. **f** CsbZIP1 interaction with CsMYB12 in a Y2H assay. **g** CsbZIP1 transactivated the promoter of *CsMYB12* in a luciferase reporter assay. **h** CsbZIP1 transactivated the promoter of *CsFLS* in a luciferase reporter assay. Differences between SD and LT (control) were analyzed. Data are from three independent experiments and are expressed as the means ± S.D. (*n* = 3). The differences in comparison with the control were analyzed by two-tailed Student’s *t* test, *p < 0.05; **p < 0.01

We next analyzed the transcriptome data of tea plant leaves under shading treatment and found that several bZIP TFs were downregulated, including three HY5 homologs, TEA012075, TEA014348, and TEA032623 (Fig. S10). However, the bZIP gene most significantly downregulated following shading is a nonannotated transcript in the tea plant genome^46^. We cloned it and found that it shared 72.62% similarity with Arabidopsis HY5 at the amino acid sequence level; therefore, we named it HY5-like TF CsbZIP1 (Fig. S11). Phylogenetic analysis revealed that CsbZIP1 clustered together with VvHY5 but apart from three other HY5 orthologs, AtHY5, HaHY5, and AaHY5 (Fig. 4a). Furthermore, *CsbZIP1* transcript levels were repressed by shading treatment (Fig. 4b). qRT-PCR analysis results showed that *CsbZIP1* was expressed at higher levels in the first, second and third leaves than in the buds, flowers, stems, fruits and roots (Fig. 4c). GFP-CsbZIP1 fusion protein signals in tobacco epidermal cells were observed in the nucleus, suggesting its nuclear localization as a TF (Fig. 4d). Moreover, Y1H assays revealed that CsbZIP1 could bind to the promoters of *CsMYB12* and two flavonol biosynthetic genes, *CsFLS* and *CsUGT78A14* (Fig. 4e). Furthermore, CsMYB12 and CsbZIP1 physically interacted with one another in a yeast two-hybrid assay (Fig. 4f), indicating a possible synergistic activation effect on *CsFLS, CsUGT78A14*, and other genes associated with flavonol biosynthesis. Furthermore, a transactivation assay revealed that CsbZIP1 bound to the promoters of *CsMYB12* and *CsFLS* and markedly activated *proCsMYB12* and *CsFLS* expression in a transactivation assay (Fig. 4g, h). These results suggested that CsbZIP1 bound directly to the *CsMYB12* promoter via the C region that contained the G-box to indirectly regulate flavonol biosynthesis in tea plants.

### R2R3-MYB repressors mediated the shading treatment-repression of flavonol synthesis

**Fig. 5:**
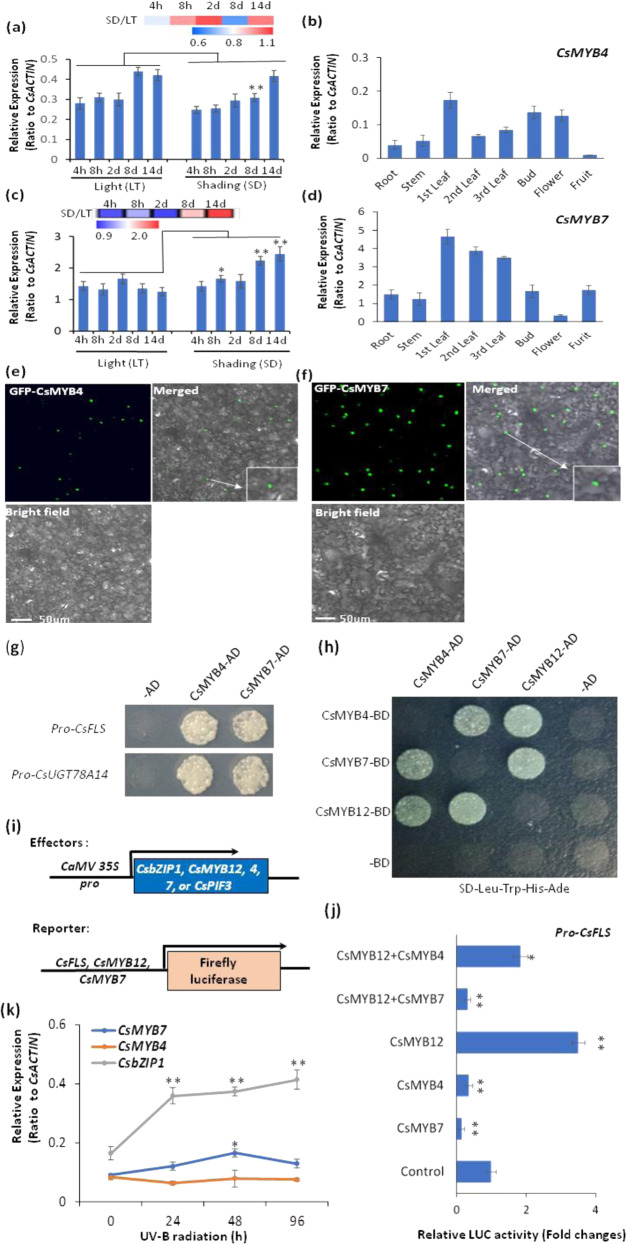
CsMYB4 and CsMYB7 repressors mediated shading-induced repression of flavonol biosynthesis. **a**, **c** Effect of shading (SD) on *CsMYB4* and *CsMYB7* expression in tea leaves compared with regular light (LT). **b**, **d** qRT-PCR data show the expression profiles of *CsMYB4* and *CsMYB7* in different stages. **e**, **f** Nuclear localization of GFP-CsMYB4 and GFP-CsMYB7 fusion in tobacco leaf epidermal cells. Bar = 50 µm. **g** Binding of CsMYB4 and CsMYB7 to the promoters of *CsFLS* and *CsUGT78A14* in Y1H assays. **h** Interactions among CsMYB12, CsMYB4, and CsMYB7 in Y2H assays. **i** Constructions of reporter and effector expression vectors for dual luciferase assays. **j** Transactivation of CsMYB12, CsMYB4, and CsMYB7 individually or in combination on the promoter activity of *CsFLS* in the luciferase reporter assay. **k** Effect of UV-B radiation on the expression of *CsMYB4, CsMYB7*, and *CsbZIP1*. Differences between shading (SD) and light (LT) (control) were analyzed. Data were from three independent experiments and expressed as the means ± S.D. (*n* = 3). Differences comparison with the control were analyzed by two-tailed Student’s *t* test, *p < 0.05; **p < 0.01

During the analysis of the transcriptome data from several light-treatment experiments^8–11^ we observed that two other R2R3-MYB TFs, *CsMYB4* and *CsMYB7*, could be markedly activated by shading treatment (Fig. 5a, c). Both CsMYB4 and CsMYB7 clustered together with VvMYBC2-L1, PtoMYB156, and other R2R3-MYB repressors in our sequence phylogeny (Fig. S12). Furthermore, both CsMYB4 and CsMYB7 repressors contain a conserved LxLxL sequence within the C-terminal region (Fig. S13). We next tested whether these proteins acted as negative regulators of flavonol synthesis during shading- or light treatment-modified flavonol biosynthesis. Both *CsMYB4* and *CsMYB7* were expressed in green tissues in tea plants (Fig. 5b, d), and both CsMYB4 and CsMYB7 were localized to the nuclei, as shown by GFP-CsMYB4 and GFP-CsMYB7 fusion expression in tobacco leaf epidermal cells (Fig. 5e, f). Furthermore, they also bound to the promoters of the *CsFLS* and *CsUGT78A14* genes, suggesting that they could regulate flavonol synthesis (Fig. 5g). The Y2H experiment results revealed that CsMYB12 interacted with MYB4 and MYB7 and that CsMYB4 and CsMYB7 interacted with each other (Figs. 5h and S14). Using the dual luciferase reporter gene system, 0800-LUC vectors of *proCsFLS*, as well as p2GW7 vectors of *CsMYB4, CsMYB7, CsbZIP1*, and *CsMYB12*, were constructed and transferred into *Arabidopsis thaliana* for promoter activation experiments (Fig. 5i). Transactivation assays with *proCsFLS*-driven LUC reporters showed that while CsMYB12 and CsbZIP1-activated *proCsFLS*, *CsMYB7*, or *CsMYB4* individually or together synergistically repressed the CsMYB12- or CsMYB12 + CsbZIP1-activated *proCsFLS*. From these analyses, CsMYB7 appeared to have stronger repression activity than CsMYB4 (Figs. 5j and S15). To further understand how these MYB TF genes respond to UV-B radiation, we also examined *CsMYB7* and *CsMYB4* expression in UV-B radiation experiments (Fig. 5k). Indeed, *CsbZIP1* was significantly upregulated by UV-B radiation, *CsMYB4* and *CsMYB7* were less changed, and only *CsMYB7* was upregulated at 48 h after radiation (Fig. 5k).

### CsPIF3 activated *CsMYB7* and thereby repressed flavonol synthesis

**Fig. 6:**
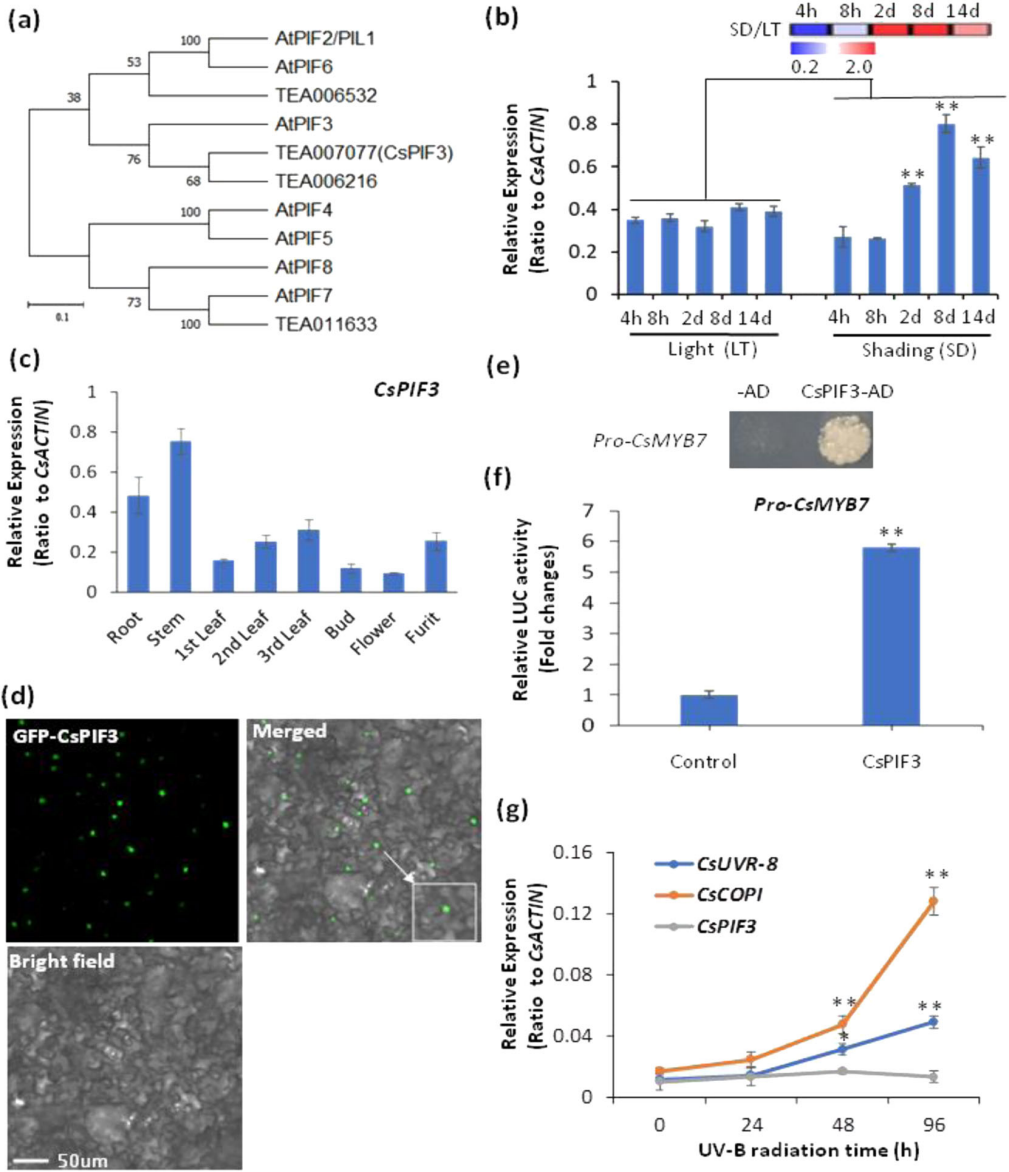
CsPIF3 activated CsMYB7, through which CsPIF3 repressed flavonol synthesis. **a** A phylogenetic tree generated by MEGA analysis using amino acid sequences of CsPIF3 and other bHLH members. *Arabidopsis thaliana* PIFs: AtPIF2, AtPIF6, AtPIF3, AtPIF4, AtPIF5, AtPIF8, and AtPIF7. **b** The shading effects on *CsPIF3* expression in tea leaves analyzed by qRT-PCR. **c** qRT-PCR data showing the expression profiles of *CsPIF3* in different stages. **d** Nuclear localization of GFP-CsPIF3 fusion protein in the leaf epidermal cells of *Nicotiana benthamiana*. Bar = 50 µm. **e** Binding of CsPIF3 to promoters of *CsMYB7* in Y1H assays. **f** Transactivation of CsPIF3 activity on *CsMYB7* with the luciferase reporter assay. **g** Effect of UV-B radiation on the expression of *CsUVR8, CsCOP1*, and *CsPIF3*. Differences between shading treatment (SD) and light (LT) control were analyzed. Data were from three independent experiments and expressed as the means ± S.D. (*n* = 3). Differences in comparison with the control were analyzed in two-tailed Student’s *t* test, *p < 0.05; **p < 0.01, *p < 0.05; **p < 0.01

We further asked how *CsMYB7* and *CsMYB4* in tea plant shoot tips were activated by shading treatment. Since *CsPIF3* genes have been shown to be upregulated by shading, we examined whether this essential light signaling gene can activate *CsMYB7* and *CsMYB4*. Of the two Arabidopsis AtPIF3 homologs, TEA006216 and TEA007077 (Fig. 6a), only the latter was dramatically upregulated by shading (Fig. S16). We thus named it *CsPIF3*. CsPIF3, AtPIF1, AtPIF3, and AtPIF8 all had conserved APB and APA elements (Fig. S17), and qRT-PCR results showed that *CsPIF3* could be upregulated in tea plant leaves by shading treatment compared with the control (Fig. 6b). *CsPIF3* displayed higher expression levels in stems, roots, and leaves (Figs. 6c and S16). Because the *CsMYB4* promoter was not assembled in the reference tea plant genome^46^, we cloned only the *CsMYB7* promoter, which contained a G-box cis-element that is reported as a binding site by AtPIF3 (Fig. S18). CsPIF3 was localized to nuclei, as shown by GFP-CsPIF3 fusions expressed in tobacco leaf epidermal cells (Fig. 6d). Y1H experiments showed that CsPIF3 can bind to and activate the promoter of the *CsMYB7* gene (Fig. 6e). Additionally, as a nucleus-localized TF, *CsPIF3* activated the *CsMYB7* promoter in a transactivation assay (Fig. 6f). These results showed that under shading, *CsPIF3* could activate *CsMYB7*, through which *CsPIF3* repressed flavonol synthesis. Under UV-B radiation, *CsUVR8* expression was slightly upregulated, and *CsCOP1* was significantly upregulated by UV-B radiation. However, in contrast to *CsbZIP1*, *CsPIF3* expression was almost unchanged by UV-B radiation (Fig. 6g).

### Suppression of *CsMYB12* and *CsbZIP1* in shoot tips affected flavonol biosynthesis

**Fig. 7:**
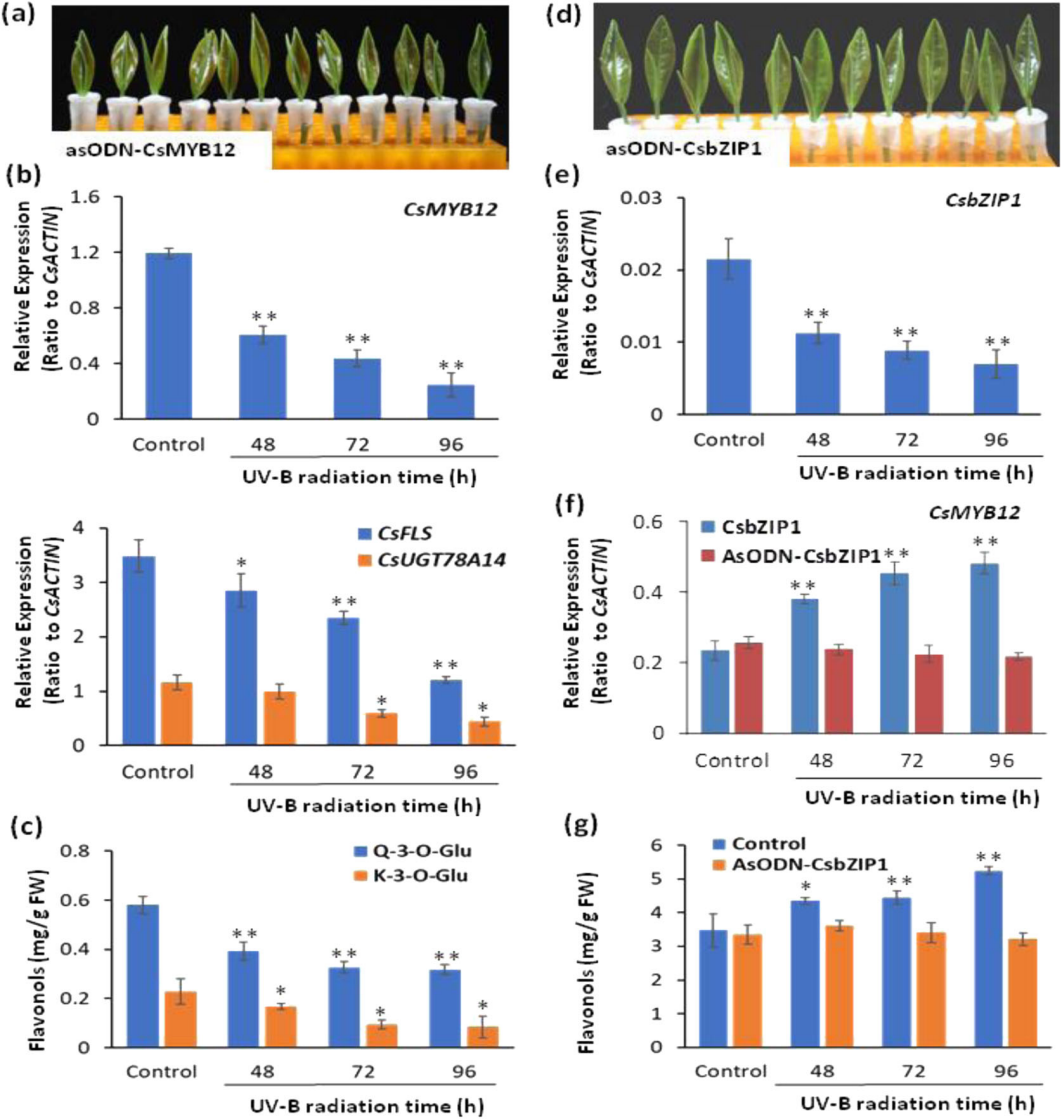
Suppression of *CsMYB12* and *CsbZIP1* in tea plant shoot tips affected flavonol production. **a** Tea plant shoot tips fed with asODN-*CsMYB12*. **b** Expression of *CsFLS, CsUGT78A14*, and *CsMYB12* in the shoot tips of tea plants. **c** Contents of flavonols after asODN-*CsMYB12* treatment. **d** Tea plant shoot tips fed with asODN-*CsbZIP1*. **e** Repression of *CsbZIP1* transcript levels in tea plant shoot tips by asODN-*CsbZIP1* treatment. **f** Changes in *CsMYB12* transcripts with asODN-*CsbZIP1* treatment upon UV-B radiation. **g** Changes in flavonol contents in asODN-*CsbZIP1* in comparison with sODN treatment. Differences between asODN treatments and sODN treatments (control) were analyzed. Data are expressed as means ± s.d. from at least three replicates. Differences in comparison with the control were analyzed in two-tailed Student’s *t* tests, *p < 0.05; **p < 0.01

To further analyze the physiological role of *CsMYB12* as a regulator of flavonol biosynthesis in tea plants, the expression level of *CsMYB12* was suppressed in the *C. sinensis* bud and 1st leaves by using an antisense oligodeoxynucleotide (asOND)-interfering gene-specific suppression strategy (Fig. 7a)^45^. asODN knockdown resulted in *CsMYB12* and two structural genes of the flavonol biosynthetic pathway, *CsFLS* and *CsUGT78A14*, which were markedly repressed over the treatment period, as verified by qRT-PCR (Fig. 7b). The contents of K-3-*O*-Glu and Q-3-*O*-Glu in the apical bud and 1st leaf were reduced by up to 1.5-fold compared with those in the control (Fig. 7c). Furthermore, *CsbZIP1* was knocked down by using a similar asODN approach to understand its regulation of *CsMYB12* following UV-B treatment (Fig. 7d). Obvious asODN suppression of *CsbZIP1* was observed only under UV radiation (Fig. 7e). In general, *CsMYB12* transcripts did not fluctuate when *CsbZIP1* was repressed under normal light intensity. However, UV-B treatment resulted in a significant upregulation of *CsMYB12* in the untreated asODN control, while *CsMYB12* was unchanged in asODN-CsbZIP1-treated shoot tips (Fig. 7f). Correspondingly, the flavonol contents did not change in asODN-bZIP1 compared with the control, in which flavonoid contents increased upon UV-B radiation (Fig. 7g). Thus, CsMYB12 appears to play a key role in the regulation of flavonol biosynthesis in tea plants. Moreover, CsbZIP1 regulates UV-B-stimulated *CsMYB12* expression and flavonol biosynthesis.

### Responsive expression of CsMYB4, CsMYB7, CsMYB12, CsbZIP1, CsUVR8, CsCOP1, and CsPIF3

To better understand the functions of *CsMYB4*, *CsMYB7*, *CsMYB12*, *CsbZIP1*, *CsUVR8, CsCOP1*, and *CsPIF3* in tea plants, we analyzed their expression patterns in different tissues or in response to UV-B, cold and salt stresses or PEG and MeJA treatments. Under UV-B treatments, *CsMYB12*, *CsbZIP1*, *CsUVR8*, and *CsCOP1* were upregulated (Figs. 1d, 5k, and 6g). However, *CsMYB4* was repressed under UV-B treatment (Fig. 5k). *CsMYB7* and *CsPIF3* were initially slightly induced and then repressed by UV-B, when *CsMYB12, CsbZIP1*, and *CsUVR8* transcripts reached the highest levels (Figs. 1e, 5k and 6g). These results are similar to those of previous studies^10^.

Other studies showed that flavonol contents in tea plant tissues also increased under MeJA treatment and salinity and PEG stresses, coupled with significant changes in the structural genes involved in flavonol biosynthesis^40,41^ (Fig. S19). *CsMYB12* and *CsMYB7* were generally more highly expressed in leaves than in roots (Figs. 3b and 5d); however, *CsMYB7* was expressed at low levels following cold and MeJA treatments (Fig. S20a, c). *CsMYB12* expression was induced by cold and MeJA treatment of leaves (Fig. S20a, c) but repressed by salt and PEG treatment (osmotic stress; Fig. S20b, d)^40,41^. Meanwhile, *CsPIF3* and *CsMYB4* showed the opposite behavior; *CsPIF3* was repressed by MeJA treatment, while CsMYB4 was initially slightly induced and then repressed by MeJA when *CsMYB12* transcripts reached their highest levels (Fig. S20c)^41^. These results indicate that light and abiotic stress regulation of flavonol synthesis and accumulation occurs at the level of transcription.

## Discussion

Characteristics of tea, such as color, taste, smell, and levels of health-conferring metabolites, are regarded as the major tea quality parameters that guide tea plant cultivation, breeding, and tea processing. These qualities depend primarily on the types and contents of tea plant-specific secondary metabolites present in the fresh tea plant leaves and the ways these starting materials are processed into teas^5^. Flavonol glycosides, such as myricetin 3-*O*-galactoside and quercetin-3-*O*-rutinoside, although present at relatively low levels in tea plant leaves compared with catechins and caffeine, have recently been recognized as among the major contributors to the bitter and astringent tastes of tea^47,48^. Therefore, their biosynthesis and regulation in tea plant leaves have been the focus of considerable attention. *FLS* and *UGT* are two critical and specific genes involved in flavonol glycoside biosynthesis^6,49^. While flavonol glycosides present in tea plant leaves grown under strong light in the spring-summer season are the major contributors to teas with stronger bitter tastes^7^, a reduction in light radiation by various measures, such as shading, has been shown to effectively improve tea quality^8,11^. Studies have revealed the biosynthetic pathways and enzymes involved in the production of a wide array of flavonol glycosides that are present at significant levels in strong light-radiated plant leaves or fruits^50,51^. Despite the fact that the regulatory mechanism underlying the high light or UV-B-radiation induction of flavonol glycoside biosynthesis is well known^51^, the repression of this activity by shading treatment is poorly understood. Significant progress has been made in understanding plant responses to high light or UV-B and shading treatments, including the characterization of several photoreceptors, *COPIs*, *HY5*, and *PIFs*, and other downstream effectors^52^. Our current study attempted to elucidate the transcriptional regulatory mechanisms underlying light-regulated flavonol accumulation in tea plants, providing new insights into the complex regulatory network controlling light- and shading-regulated flavonol biosynthesis.

### High light or UV-B radiation regulated flavonol biosynthesis in tea plant leaves

**Fig. 8:**
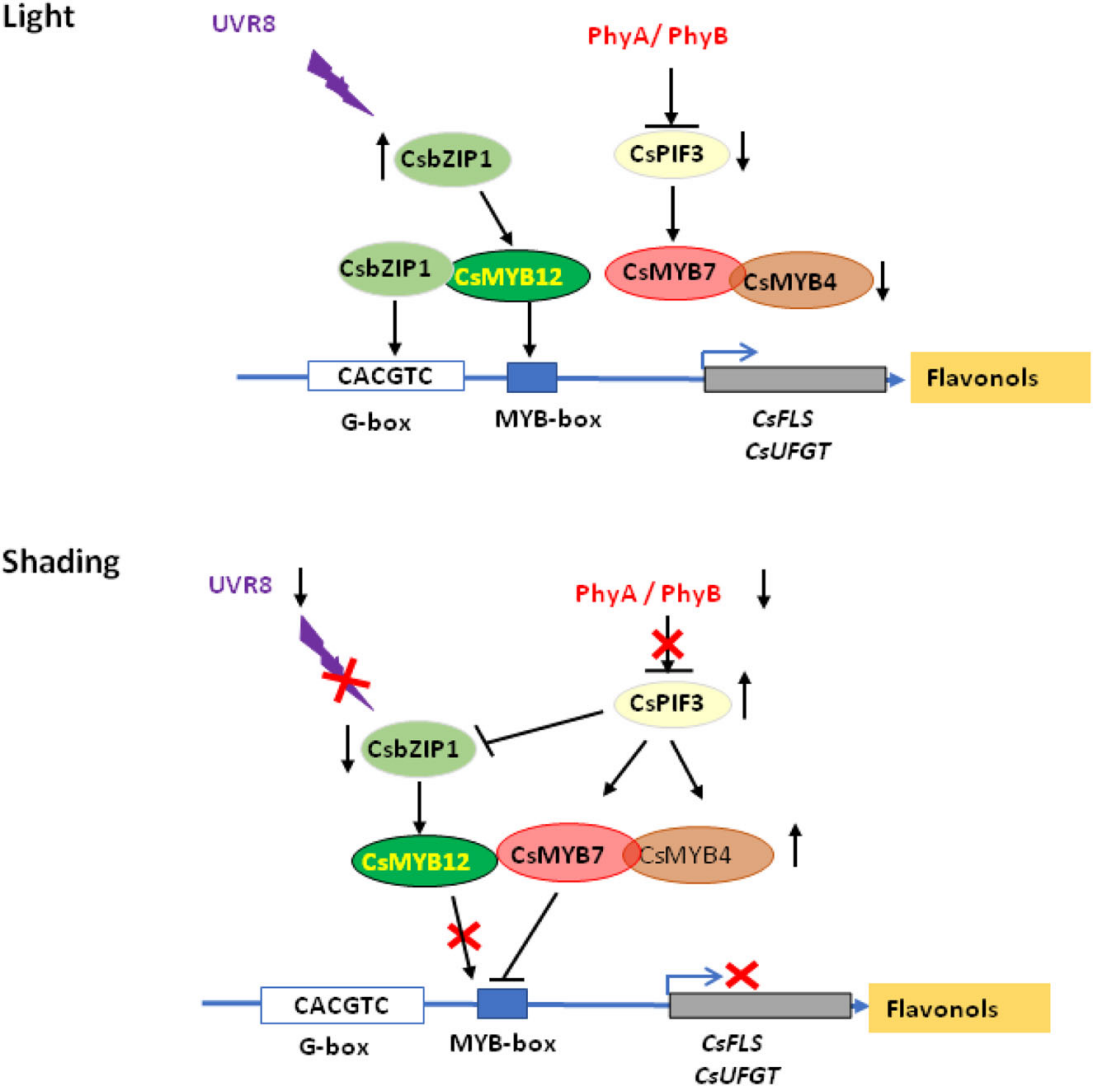
A proposed model for light- and shading-mediated flavonol biosynthesis in tea plants. Under high light conditions, UVR8 activates *CsbZIP1*, which enhances *CsMYB12* transcription and activity. Both CsbZIP1 and CsYMB12 synergistically activate *CsFLS* and *CsUFGT* expression and promote bitter-tasting flavonol biosynthesis and accumulation, thereby decreasing the processing suitability of tea plant leaves. Meanwhile, *CsPIF3, CsMYB7*, and *CsMYB4* are expressed at low levels and inactivated. However, under shading conditions, *CsbZIP1* is repressed, while *CsPIF3*, *CsMYB7*, and *CsMYB4* are activated. The repression of CsPIF3 by PhyA and PhyB is relieved, allowing CsPIF3 to interact with and repress CsbZIP1 activity. Shading upregulates the expression of the repressor genes *CsMYB7* and *CsMYB4*. CsMYB7 and CsMYB4 can interact with CsMYB12 and thus interfere with CsMYB12 activation of flavonol synthesis. CsMYB7 and 4 also directly repressed *CsFLS* expression and, as a consequence, flavonol biosynthesis under shading conditions

Many reports have investigated tea metabolites under shade or altered light conditions. Previous studies have focused predominantly on the effect of shading treatment on catechin biosynthesis in tea cultivars^8,11^. In the present study, we found that flavonols decreased even more significantly in tea leaves than catechins did upon shading treatment (Fig. 2b). Consistent with these transcriptome data, our qRT-PCR data showed that the expression of key flavonol pathway genes, including *CsF3H*, *CsF3*′*H*, *CsF3*′*5*′*H*, *CsFLS*, and *CsUGT78A14*, was markedly reduced by shading treatment and conversely was upregulated by UV-B. We further revealed that the MYB TF *CsMYB12* directly regulated these flavonol synthesis genes and that two light signaling TFs, *CsbZIP1* and *CsPIF3*, worked upstream of *CsMYB12*, thereby acting in concert to translate UV-B and high-light radiation or shading treatment into effects on flavonol biosynthesis. Furthermore, we uncovered an even more complex regulatory network by naturally or deliberately regulating lighting or shading treatments. These studies likely reveal the two sides of the same coin. That is, UV-B or high-light radiation induces *CsbZIP1* and *CsMYB12* and dominantly upregulates flavonol synthetic genes, thereby promoting the accumulation of bitter-tasting flavonols in tea plant leaves in the spring-summer season, whereas under shading treatment, both *CsbZIP1* and *CsMYB12* are repressed as the result of both COP1-mediated CsbZIP1 degradation and the regulation of two R2R3 repressors, *CsMYB7* and *CsMYB4*, by another bHLH light signaling protein (CsPIF3) to further effectively repress *CsMYB12* activity and thus repress flavonol biosynthesis (Fig. 8). Furthermore, upregulated *CsPIF3, CsMYB7*, and *CsMYB4* mediate red- or far-red light signaling in darkness. Thus, it is suggested that shading treatment, similar to red light radiation, can effectively reduce the biosynthesis of flavonols and that two mechanisms explain the reduced flavonol content in tea plant leaves under shading treatment.

### MYB activators or repressors as regulators of phenylpropanoid metabolism in plants

Many R2R3-MYB activators of flavonol biosynthesis have been characterized. AtMYB11, 12, and 111 control flavonol accumulation in different parts of the Arabidopsis seedling^18^. VvMYBF1 was confirmed to complement the flavonol-deficient phenotype of the *AtMYB12* mutant^20^. In addition, under abiotic stress conditions, MYB repressor TFs are particularly important, and R2R3-MYB repressors contain a conserved LxLxL sequence within the C-terminal region^53^. In grapevine, three flavonoid repressor MYBs, namely, MYBC2-L1, MYBC2-L2, and MYBC2-L3, were identified^22^. CsMYB4a, as a lignin synthesis repressor, was identified in tea plants^54^. Therefore, activator–repressor systems coordinate the fine tuning of critical metabolite biosynthesis and accumulation^22^. Subgroup 4 of R2R3-MYB transcription factors in Arabidopsis consists of repressors MYB3, MYB4, MYB7, and MYB32, possessing the conserved EAR repression motif^54^. In this study, we isolated three potential genes, *CsMYB12*, *CsMYB4*, and *CsMYB7*, from tea plants that were hypothesized to positively and negatively regulate flavonol biosynthesis. Then, overexpression of *CsMYB12* promoted the accumulation of flavonol in soybean roots (Fig. S9). The luciferase reporter assay results showed that *CsMYB4* and *CsMYB7* had a significant effect on the negative regulator *CsFLS* (Fig. 5j). Thus, *CsMYB4* and *CsMYB7* can affect flavonol biosynthesis, rendering them repressors with potentially broad impacts on tea plant secondary metabolism. It seems likely that the coordinated action of repressor and activator MYBs could be important for the fine tuning of flavonoid biosynthesis during development or following stress.

### CsbZIP1 and CsPIFs differentially regulate flavonol synthesis under normal light and shading conditions

Light is one of the most important environmental factors regulating flavonoid biosynthesis^55^. HY5 and activator MYB TFs regulate flavonoid biosynthesis and are required for UV-B tolerance. Given the importance of HY5 in a broad panel of photomorphogenic responses downstream of multiple photoreceptors^26,52^, it is of interest to characterize the *CsbZIP1* gene and its signaling pathway in the context of light-induced and developmental regulation of tea plant secondary metabolism. In contrast, PIF3 is a bHLH TF with a light regulation mechanism on the other darkness side that binds to the palindromic G box motif CACGTG, which is common to many plant genes^56,57^. In this study, we functionally characterized two light signaling genes, *CsbZIP1* and *CsPIF3*, in the regulation of flavonol biosynthesis in light- and shading-treated tea plants through their binding and activating and repressing *CsMYB12*, as well as two negative MYB regulator genes, *CsMYB4* and *CsMYB7*. The complex regulatory network composed of both activators and repressors of various kinds of TFs related to the light and shading responses in tea plants can explain the increased levels of bitter-tasting flavonols under high light (including stronger UV-B radiation during the early summer and late autumn), as well as the drastic reduction in flavonol levels in tea plant leaves under shading treatment.

The interaction between CsbZIP1 and CsMYB12 and the direct binding of CsbZIP1 to the promoter of the *CsMYB12* gene for its activation play a dominant role in connecting light signal perception to flavonol biosynthesis in tea plants. Even under shading treatment, reduced *CsbZIP1* transcript levels remain a critical factor maintaining certain levels of flavonols in tea plant leaves. *CsPIF3* is expressed at significantly higher levels in tea plant leaves under shading treatment, and CsPIF3 binds to the G-box in the promoter of *CsMYB7* to upregulate the *CsMYB7* repressor and, more likely, *CsMYB4*. Both CsMYB7 and CsMYB4 repressed *CsFLS* transcription and thus interfered with CsMYB12 function as activators of *CsFLS* and other flavonol biosynthetic genes under shading treatment. The regulatory function of CsMYB12 seems highly specific to flavonoid biosynthetic genes, such as *CsFLS* and *CsUGT78A14*. However, the regulatory targets of CsMYB4 and CsMYB7 may be nonspecific, since their upregulation under shading treatment or changes in lighting could also be negatively correlated with catechin levels. Under shading treatment, catechin contents also generally decreased when tea plant shoot tips became less bitter. Further study will be needed to demonstrate how these negative MYB regulators work in the regulation of flavonoid biosynthesis in tea plants.

It is possible that AtPIF3 and HY5 interact directly; alternatively, their antagonistic effects may be mediated through another factor, such as cryptochromes and UVR8^39^. HY5 binding to the promoters of UV-B-responsive genes is enhanced by UV-B in a UVR8-dependent manner in *Arabidopsis thaliana*. In agreement with this observation, overexpression of REPRESSOR OF UV-B PHOTOMORPHOGENESIS2, a negative regulator of UVR8 function, blocks UV-B-responsive HY5 enrichment at target promoters^58^. A T/G-box in the HY5 promoter is required for its UV-B responsiveness. HY5 and its homolog HYH bind to the T/G(HY5)-box cis-acting element to activate its own expression redundantly upon UV-B exposure. HY5 and HYH interact directly with a T/G-box cis-acting element of the HY5 promoter, mediating the transcriptional activation of HY5 in response to UV-B^59^.

In summary, UV-B radiation promoted and shading repressed flavonol biosynthetic genes and consequently flavonol production in tea plant leaves. We demonstrated here that the different effects of light and shading involved CsbZIP1 and CsPIF3, a flavonol biosynthesis activator CsMYB12, and two MYB repressors CsMYB7 and CsMYB4. UV-B radiation of tea plants upregulated *CsbZIP1* and *CsMY*B12 (Figs. 1d and 5k), whereas 90% shading treatment clearly upregulated *CsCOP1* and repressed *CsbZIP1* and *CsMYB12* (Figs. 1d, 4b, and S4a). CsbZIP1 acted as an activator of *CsMYB12* and *CsFLS* and *CsUGT78A14* genes with CsMYB12 to promote UV-B-induced flavonol production. However, after shading treatment, *CsbZIP1* and *CsMYB12* were repressed to lower expression levels. Meanwhile, shading treatment activated *CsPIF3*, *CsMYB7*, and *CsMYB4* to antagonize the effect of *CsbZIP1* and repress the *CsMYB12, CsFLS*, and *CsUGT78A14* genes. Both CsMYB7 and CsMYB4 repressed *CsFLS* and *CsUGT78A14* by directly binding to their promoters. CsMYB7 and CsMYB4 also directly interact with CsMYB12 and may interfere with CsMYB12 activation activity. Furthermore, CsPIF3 activated *CsMYB7* through binding to its promoter. This study provides new insights into the mechanism of how light regulates the production of bitter-tasting flavonols in tea plants, which may provide molecular tools for the genetic improvement of tea quality and flavor.

## Supplementary information

Supplemental ures S1-S19

Supplemental Dataset S1-S4

## Data Availability

The author responsible for the distribution of materials integral to the findings presented in this article in accordance with the policy described in the Instructions for Authors is Jian Zhao (jianzhao@ahau.edu.cn).
