# Supplementary material for: CsbZIP1-CsMYB12 mediates the production of bitter-tasting flavonols in tea plants (Camellia sinensis) through a coordinated activator–repressor network
Source: Hortic Res. 2021 May 1;8:110. doi: 10.1038/s41438-021-00545-8 (PMC8087823; doi:10.1038/s41438-021-00545-8)
Supplement: Supplementary file 1 — Supplemental ures S1-S19 [file 41438_2021_545_MOESM1_ESM.pdf]

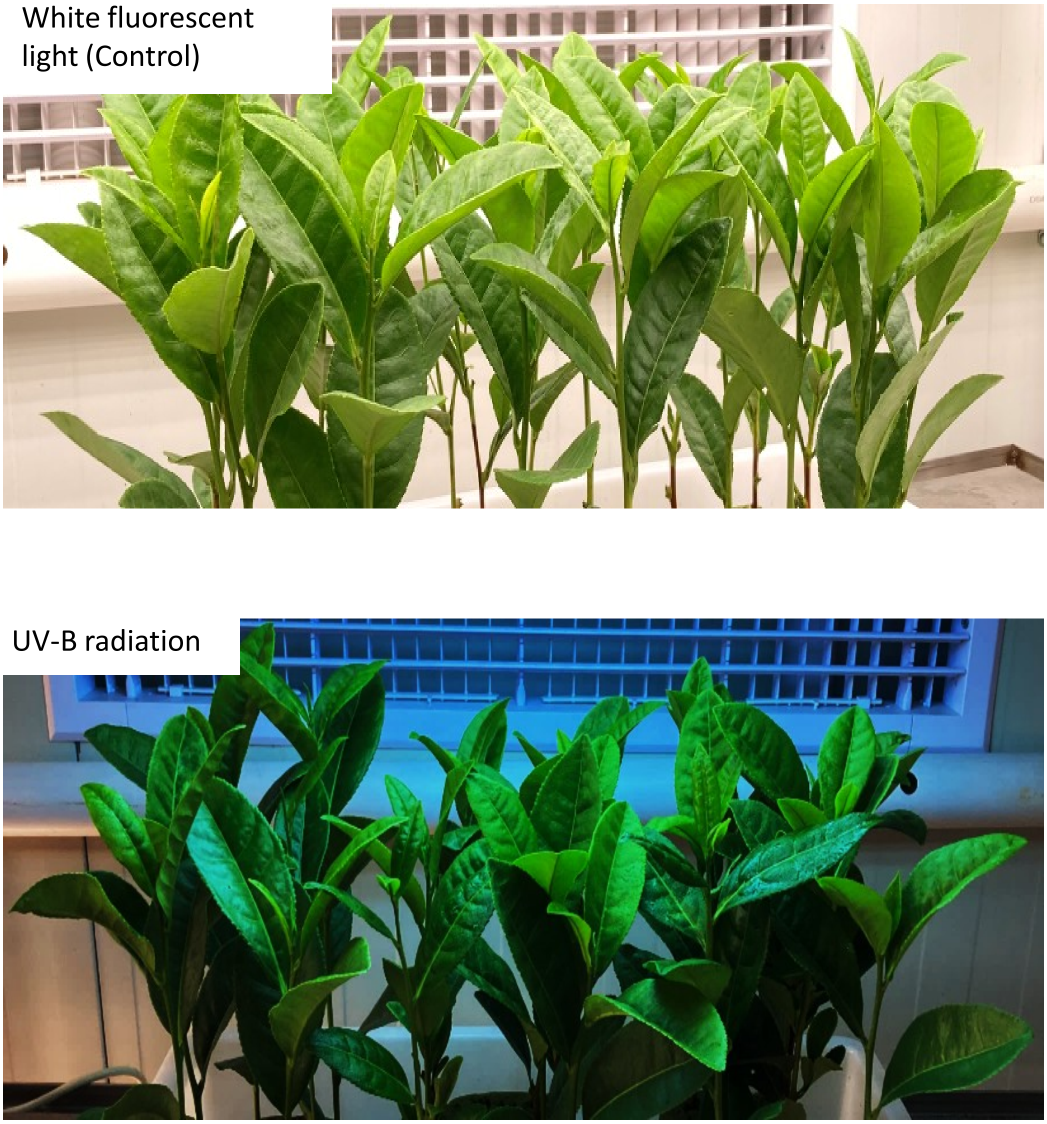


Fig S1. UV-B radiation on tea plant seedlings
UV-B conditions (300 μW•cm-2, photoperiod of 12 h per day, provided with a special lamp (PHILIPS NARROWBAND TL 20W, Poland) with characteristic peak at 311nm.25/18°C (light/dark)

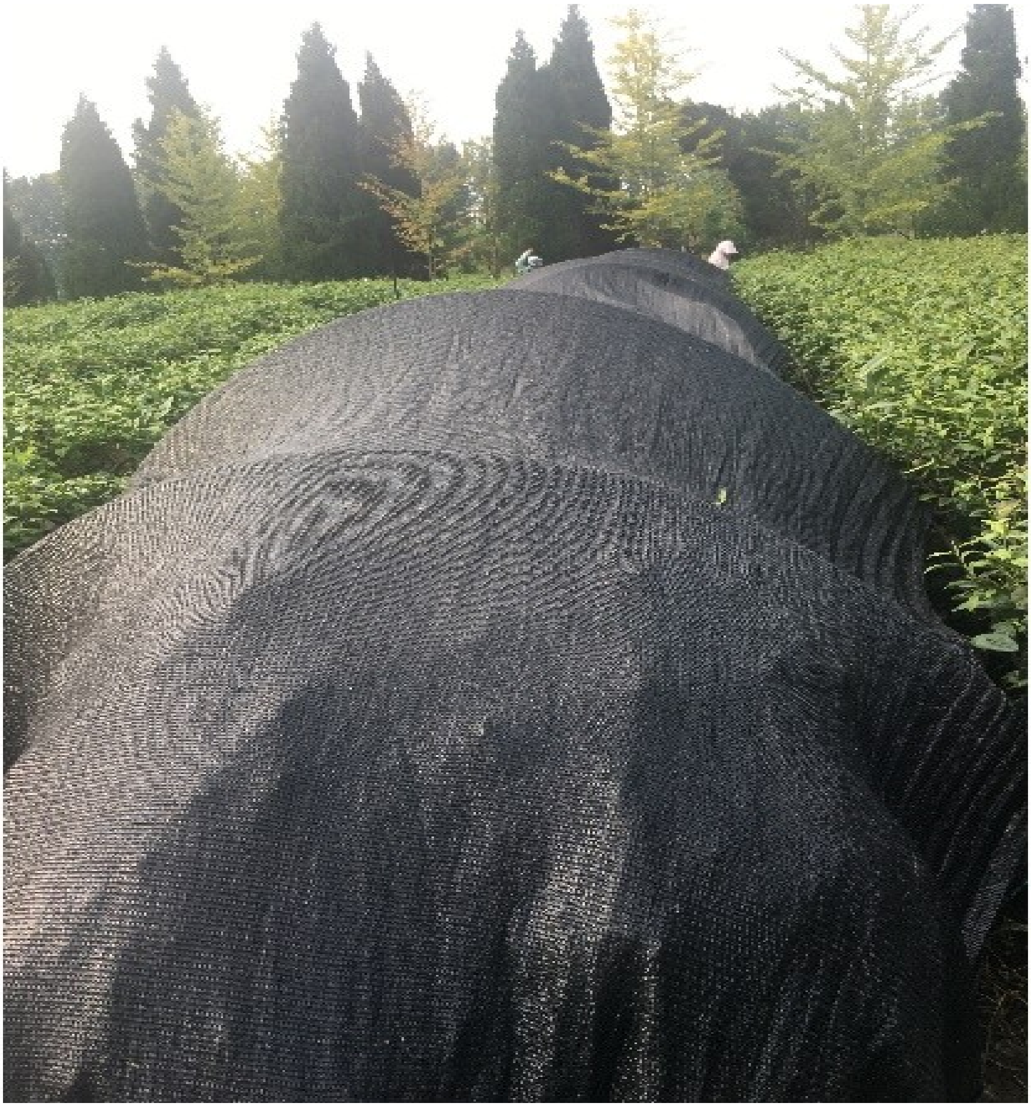

Fig S2. Shading treatment on tea plants in the tea garden.
The shading experiment was carried out in Anhui Agricultural University research tea plantation (31°. 55′ North, 117°. 12′ East; Hefei City, Anhui Province, China). 12 rows of C. sinensis cv. Shuchazao tea plants (50 m long and 1.4 m wide of each row, 2 m between and 0.6 m within row spacing) were selected for the treatments.
The tea plants were 10 years old from cuttage propagation (1.4 m wide and 1.5 m tall from the soil surface, 0.5 m between plants within the row).

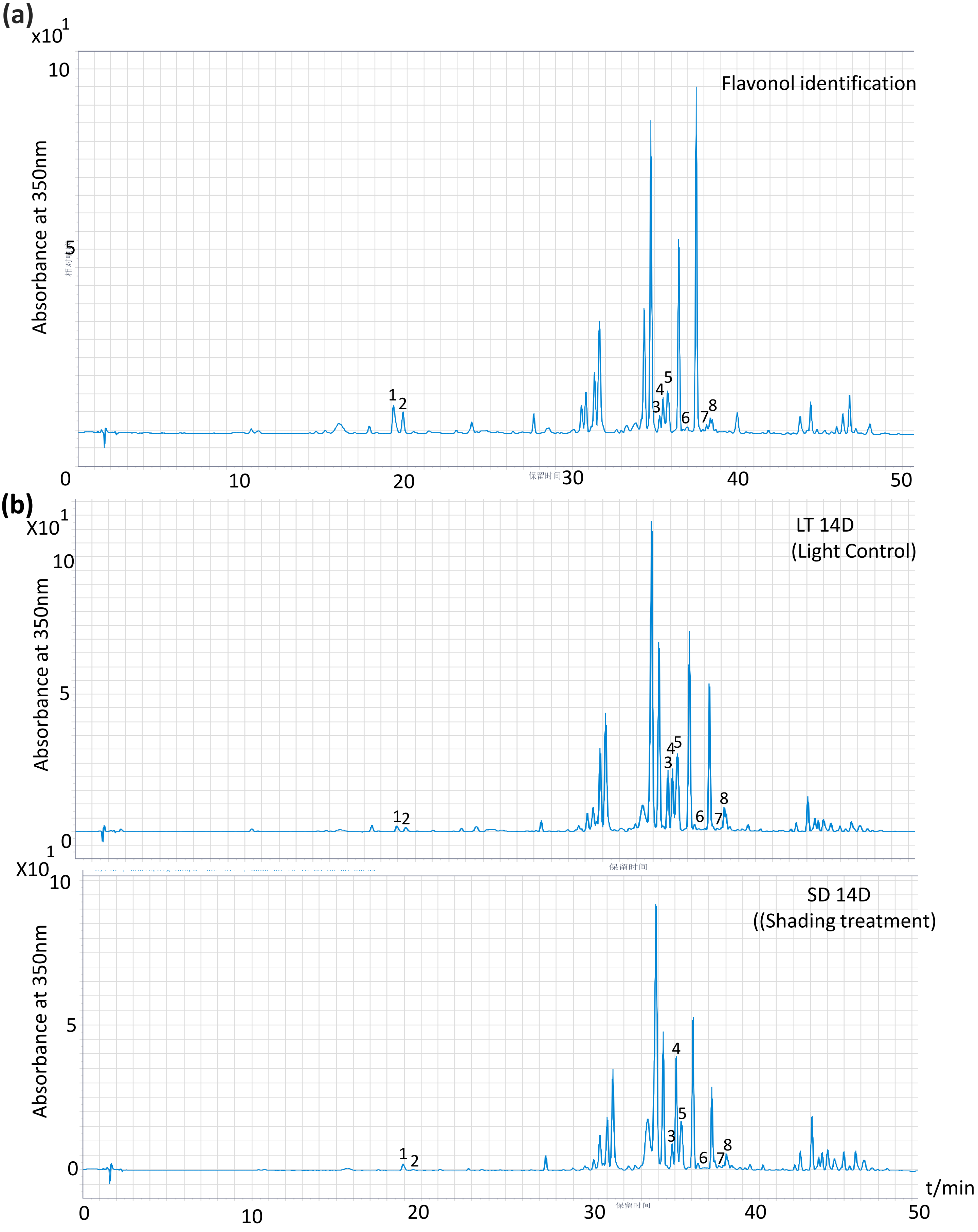
(a)
x10[1]
Absorbance at 350nm
Flavonol identification
1
2
3
4
5
6
7
8
0
10
20
30
40
50
保留时间
(b)
X10[1]
Absorbance at 350nm
LT 14D
(Light Control)
1
2
3
4
5
6
7
8
X10[1]
Absorbance at 350nm
SD 14D
((Shading treatment)
1
2
3
4
5
6
7
8
0
10
20
30
40
50
t/min
保留时间

Fig S3. Chromatogram of UPLC for phenolic compounds in tea leaves: UPLC Chromatogram at 350nm.
(a): Flavonols standards. 1：M-3-O-Gal 2：M-3-O-Glu 3：Q-3-O-Gal 4：kaempferol 3-O-galactosylrutinoside 5：Q-3-O-Glu/R 6：K-7-O-GLU 7：K-3-Gal 8：K-3-O-Glu
(b):Control and shading of flavonols were analyzed with UPLC.

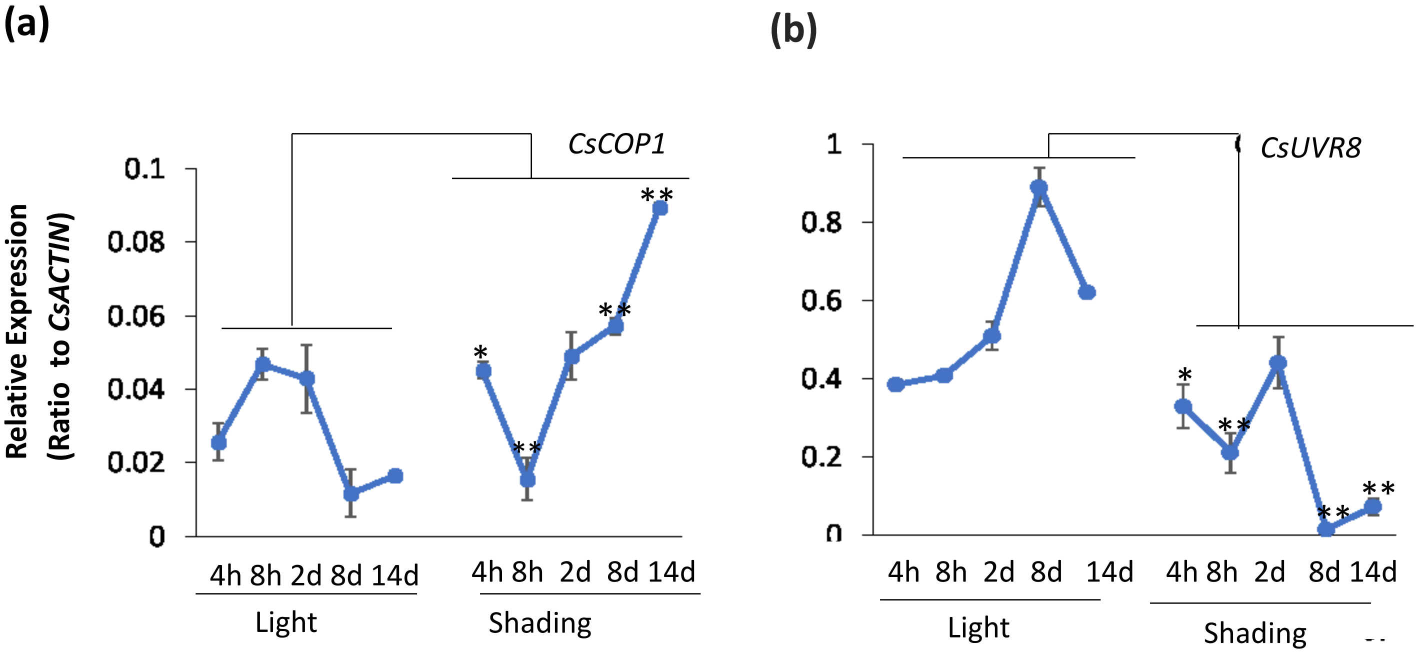


Fig S4. The shading effects *CsCOP1* and *CsUVR8* expression in tea leaves analyzed by qRT-PCR. Differences between SD and LT (control) were analyzed. Data were from three independent experiments and expressed as means±S.D. (n = 3). Differences were analyzed, *p < 0.05; **p < 0.01 in student's t-test.

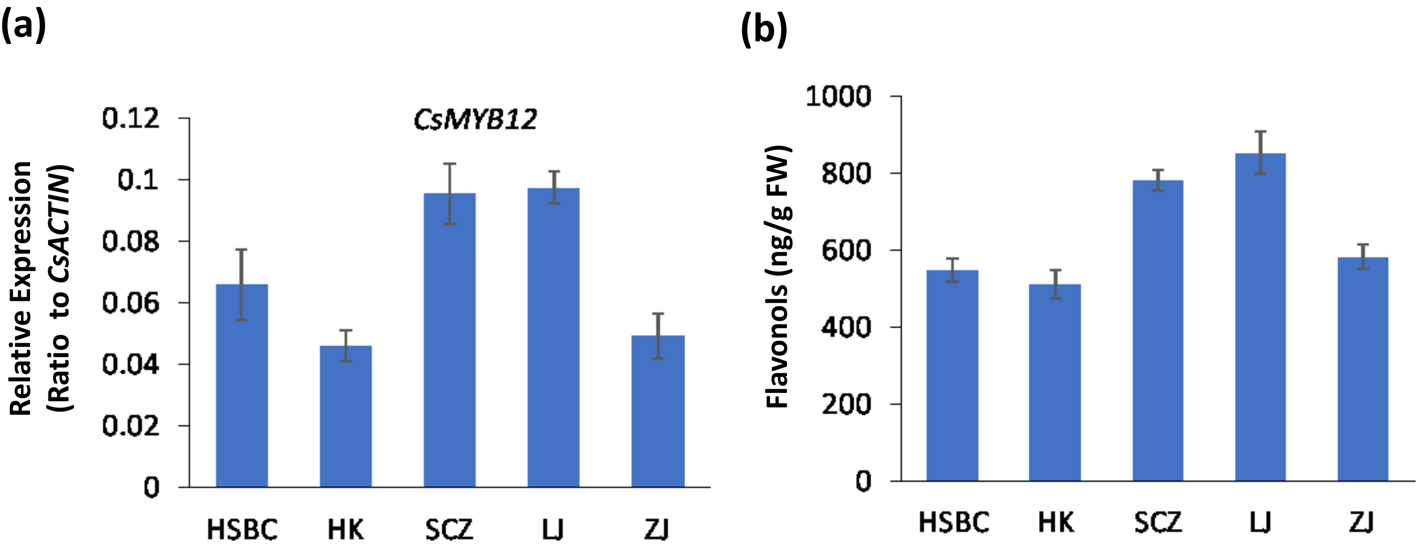


Fig S5. Relative expression level of CsMYB12 and contents of flavonoids metabolites in different tea plants

(a): Expression of CsMYB12 in different species of tea plant.

(b): Total Content of flavonols in different species of tea plants.

Long jing (LJ) ,Shu Cha Zao (SCZ) ,Huang Shan Bai Cha (HSBC), Huang Kui (HK) and Zi Juan (ZJ).

(a)

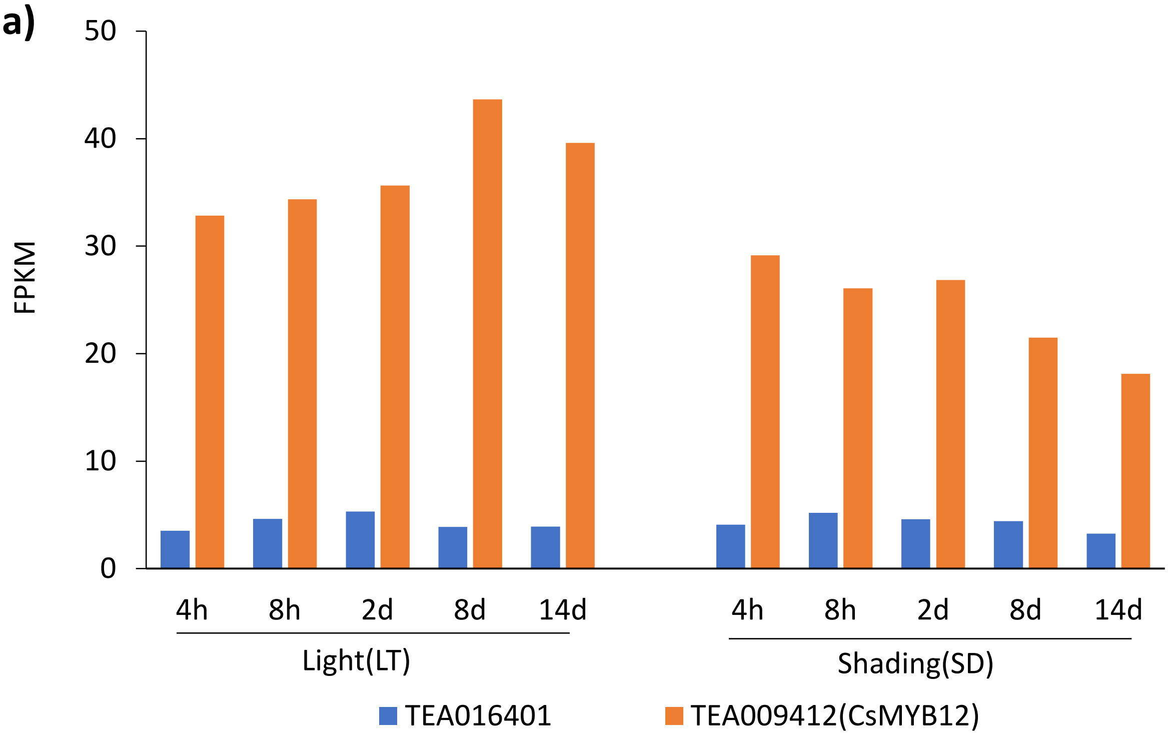


(b)

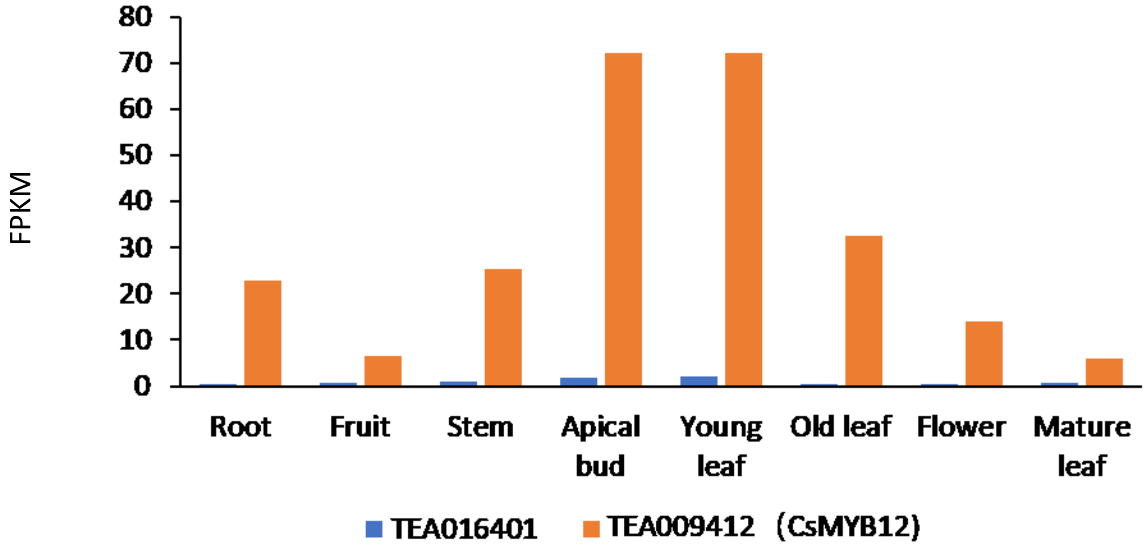


Fig S6. Expression patterns of TEA01640 and TEA009412

(a) Expression patterns of of TEA01640 and TEA009412 tea leaf under shading.

(b) Expression profiles of of TEA01640 and TEA009412 in different stages.

```
                                     R2 Domain                                    R3 Domain
CsMYB12    MGRAPCCEKVGLKRGRWTAEEDEILTKYILANGEGSWRSMPKNAGLLRCGKSCRLRWINYLRSDLKRGNISSEEEEIITKLHSTLGNRWSLIAGHLPGRTDNEIKNYWNSHLSRKIQSFR   120
AtMYB111   MGRAPCCDKIGLKRGRWTAEEDEILTNYIQTNGEGSWRSLPKKAGLLRCGKSCRLRWINYLRRDLKRGNITADEEEIIVKLHSLLGNRWSLIATHLPGRTDNEIKNYWNSHLSRKIYAFT   120
VvMYBF1    MGRAPCCEKVGLKKGRWTAEEDEVLVKYIQANGEGSWRSLPKNAGLLRCGKSCRLRWINYLRADLKRGNFSEEEEEIIIKLHASLGNRWSMIAGQLPGRTDNEIKNYWNSHLSRKVHSFR   120
SbMYBY1    MGRAPCCDKVGLKRGRWTAEEDQILANYIAEHGEGSWRSLPKNAGLLRCGKSCRLRWINYLRADVKRGNISKEEEDIIIKLHATLGNRWSLIASHFPGRTDNEIKNYWNSHLSRQIHTYR   120
MdMYB22    MGRAPCCEKVGLKKGRWTAEEDEILLNYIQANGEGSWRSLPKNAGLLRCGKSCRLRWINYLRADLKRGNISSQEEDIIIKLHASLGNRWSLIASQLPGRTDNEIKNYWNSHLSRKIGTFR   120
Consensus  mgrapcc kvglk grwtaeedeil  yi  ngegswrslpknagllrcgkscrlrwinylr dlkrgnis  ee ii klh  lgnrwslia  lpgrtdneiknywnshlsrki  fr

CsMYB12    RPCKNEAPPPMAVDLAKSTVATKRRGGRTSRSAMKKNKTQNPTKGPNPKPVKKTEPNASVPMPQTPTLQTESLSSAISWQEESVVKINLDLEGNKEGVIVGSPHHDDHAGLALCGGEERK   240
AtMYB111   A.VSGDEHN.LVVDDLVLKKSCSSSTGAKNNIKTKKKKKG.............RTSRSSMKKHKQMVTASQCFSQPKELESEIS.......DGEKNGNFEGE....SLGPYEWLDGEIER   214
VvMYBF1    R.LTNEGPS.MVIDLAKVTTAHKRKVGRTSRWAMKKNRSD........KSIREDVNKSSLEKPKGDDDGNGVIAEKETRSETMTGDLYAQVNEEENPELMAS.HLLGCGGRMFGEGSETF   229
SbMYBY1    RKYTAAPDTTVIIDMSKLHSAEKRRGGRTPGWSPKSSSAN......TTTNTTSSKTNKSKETDPGPGLKSACDAKGASSPPTAATTTSAASSPRHSDGARSAVVDPDPNQPNSSSGSTAE   234
MdMYB22    R...PATTTVITTEISTSVPPAGDEVSAAMELGPPKRRGG.............RTSRWAMKKNKTYSTTKPKGLKARLRQSHHKHDN..IAAAAADDDRLNN....AHEAIALPTKSNNK   218
Consensus  r            d          g       kk                     s                                                      g

CsMYB12    RKSEYNEGLDESGMLCFDD.IMSDELVDPKGVTILGEERENDTAVFINEE.RESDAAAVVISEERERGGGAMNMSSNGESSSTNS.YIDDWDWVWDWDAVVQGNEIWGDEEENMKFSWLW   357
AtMYB111   .......LLSGCVWECTSE..EPVIGVNDEKACESGDNSSCCVGLFEEEQ.GNDTKIGHIGITNVDDHGMKVEKEREGSVLSSTSNENNDKDWWVG..LCNSSEVGFGIDEE..LLDWEF   320
VvMYBF1    G....PFRVPEVEGLCFSENMESGVLVDGGGVVSSGTEERASGATCSN...KTTPFGGDVEGRNLSSDNGDQTVDQWPSCSSPTS.YFDDWNWES...SAVNGQELWDEKEE..MLSWLW   336
SbMYBY1    G....PCSGEDATGPWELDPIDLGDLWEAESEIDALMSMDAPLEGFDAVVGEAQAQVDDLFDMDMDWDGFAAHLWGGPEQNDHIAELQQAAEPQATAAACTPNEHEPQVAAA..AAACTP   348
MdMYB22    N.....VDTMQDGYVLLMEVPDQQQETRGGGIAMPATVIDHQKETDGEKL.ILGPHPHGHDDDDMNEYVGINGGLLGFTDYLMDDINEDEILDPNGVMALSSSEINIHQDADHFPDAVIS   332
Consensus

CsMYB12    ESDNGKGDSIVNNKLGEINCEKQEAVVAWLLS..                                                                                        389
AtMYB111   Q.....GNLTCQSVDLWDLSDIGEITLE......                                                                                        343
VvMYBF1    EDSDG.GEVECETFGGDLDCEKQDAMVAWLLS..                                                                                        367
SbMYBY1    DEHEPQAAAAAAAATCTPDEHGLEAFETWLLSDS                                                                                        382
MdMYB22    DHQDTELPSSCDQLVICPNKVMTTTTTYYGIQ..                                                                                        364
Consensus
```

Fig S7. Amino acid sequence alignment of CsMYB12 and MYB homologues from other plants

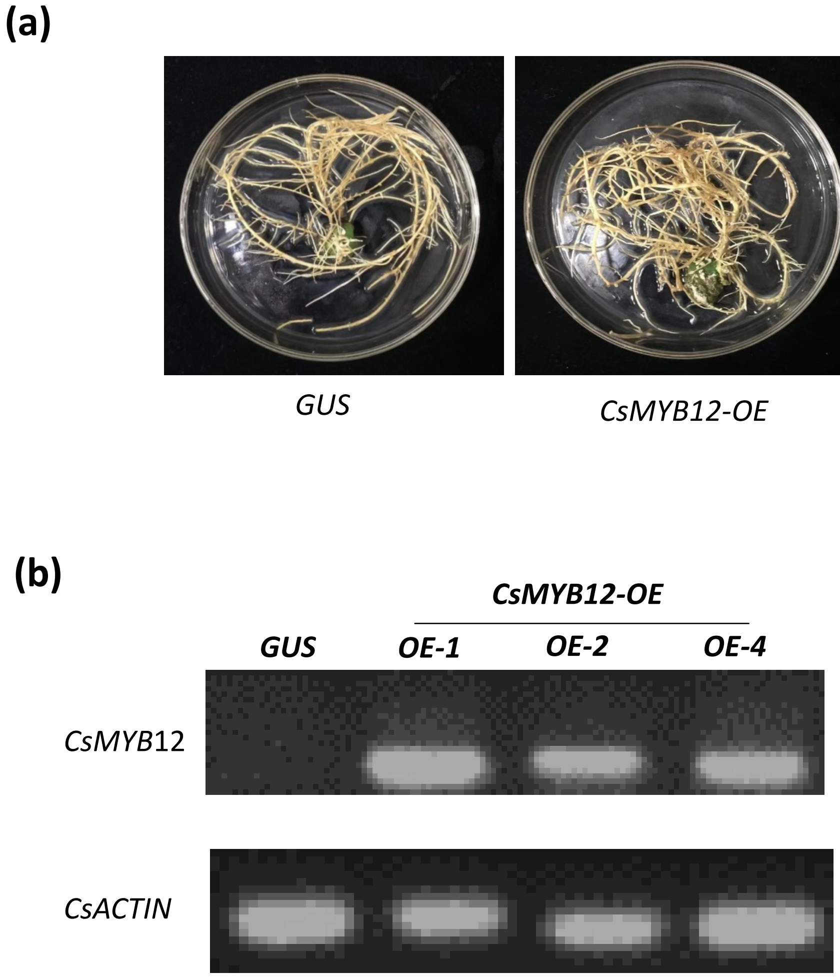


Fig S8. Generation of soybean hairy roots overexpressing *CsMYB12* or *GUS* genes.
(a) Photos of CsMYB12-OE- or GUS- overexpressing hairy roots
(b) Semi-quantitative RT-PCR examination of overexpression of CsMYB12-OE in representative transgenic hairy root lines.

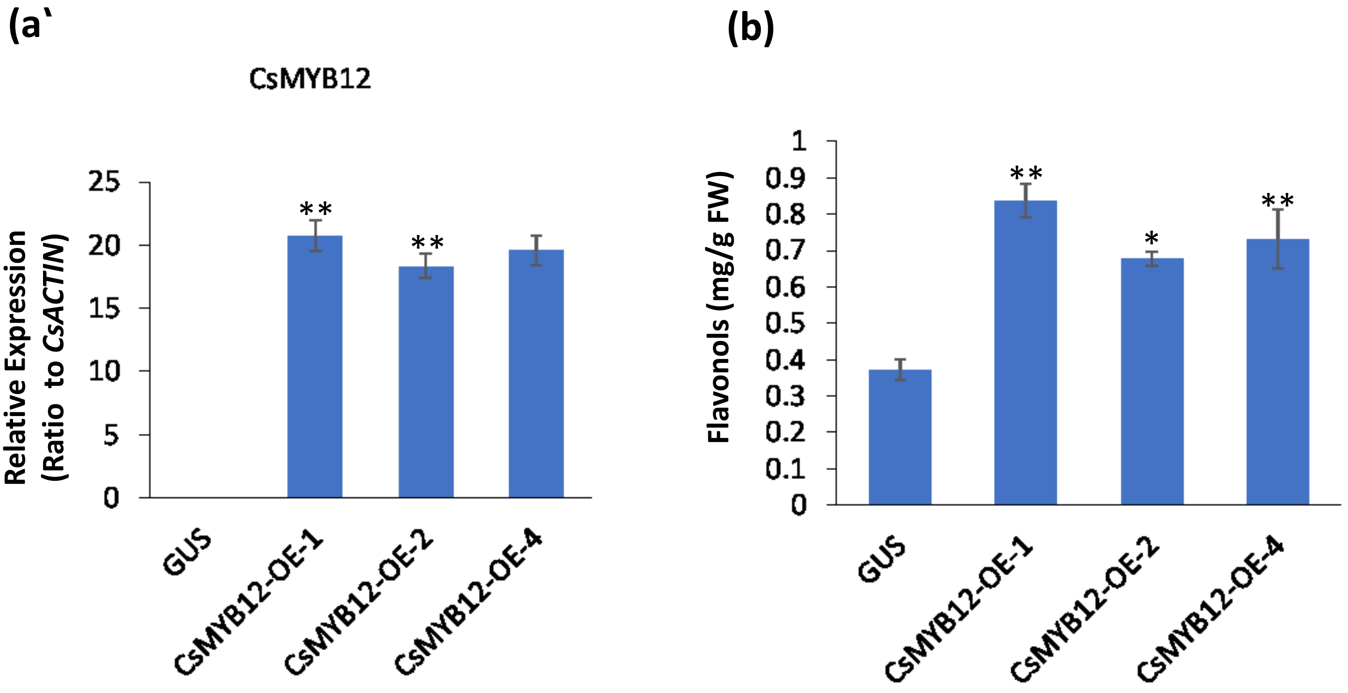


Fig S9. CsMYB12 overexpression affects flavonoids contents in hairy roots.
(a). qRT-PCR confirmation of *CsMYB12* expression in transgenic hairy root lines and GUS (control).
(b). UPLC chromatograms for flavonols profiles in *CsMYB12* overexpression hairy root lines and GUS (control).
Data were from three independent experiments and expressed as means±S.D. (n = 3). Differences were analyzed, *p < 0.05; **p < 0.01 in student's t-test.

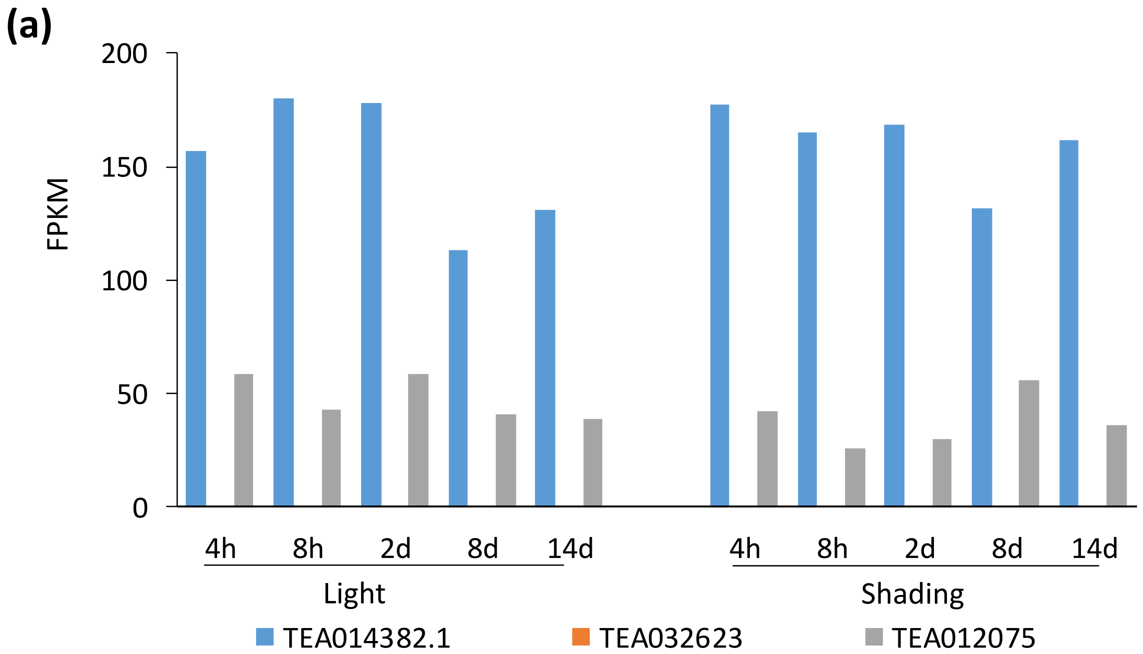


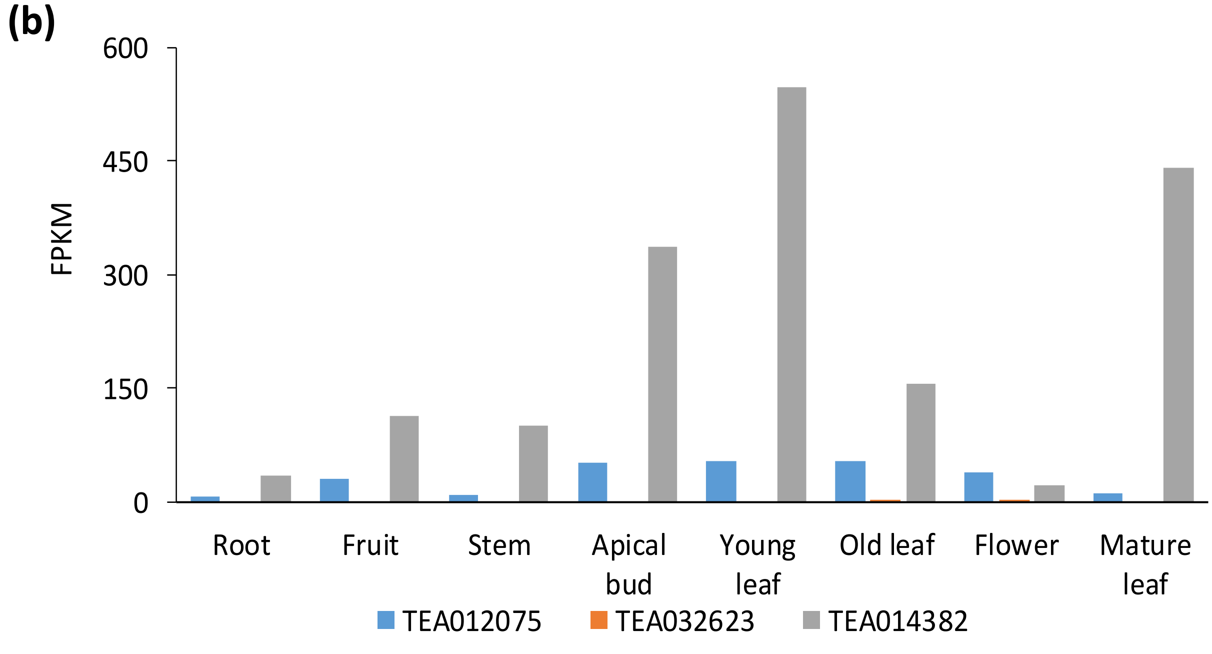


Fig S10. Expression patterns of HY5-like bZIP genes TEA012075, TEA032623 and TEA014382.
(a) Expression patterns of TEA012075, TEA032623 and TEA014382 tea leaf under shading.
(b) Expression profiles of TEA012075, TEA032623 and TEA014382 in different stages.

```
AtbZIP    MQEQATSSLAASSLPSSSERSSSSAPHLEIKEGIESDEEIRRVPEFGGEAVGKETSGRES    60
CsbZIP1   MQEQATSSIAASSLPSSSERSSSSALQLEVKEGMESDDEIRRVPEIGGEAARGSVSFREA    60
Consensus mqeqatssiaasslpssserssssalhleikegiesddeirrvpefggeaaggetsfrea

AtbZIP    GSATGQERTQATVGESQRKRGRTPAEKENKRLKRLLRNRVSAQQARERKKAYLSELENRV    120
CsbZIP1   GSTAGPDRVQASS.EGMRKRGRSPADKENKRLKRLLRNRVSAQQARERKKAYLTELEGRV    119
Consensus gsaagpdrtqassgegmrkrgrspadkenkrlkrllrnrvsaqqarerkkaylselegrv

AtbZIP    KDLENKNSELEERLSTLQNENQMLRHILKNTTGNKRGGGGGSNADAS    167
CsbZIP1   KDLEKKNSELDERVSTLQNENQMLRHILKNTTAGMQEGRK.......    159
Consensus kdlekknselderlstlqnenqmlrhilknttagkqeggggsnadas
```

Fig S11. Amino acid sequence alignment of CsbZIP1 and AtbZIP .

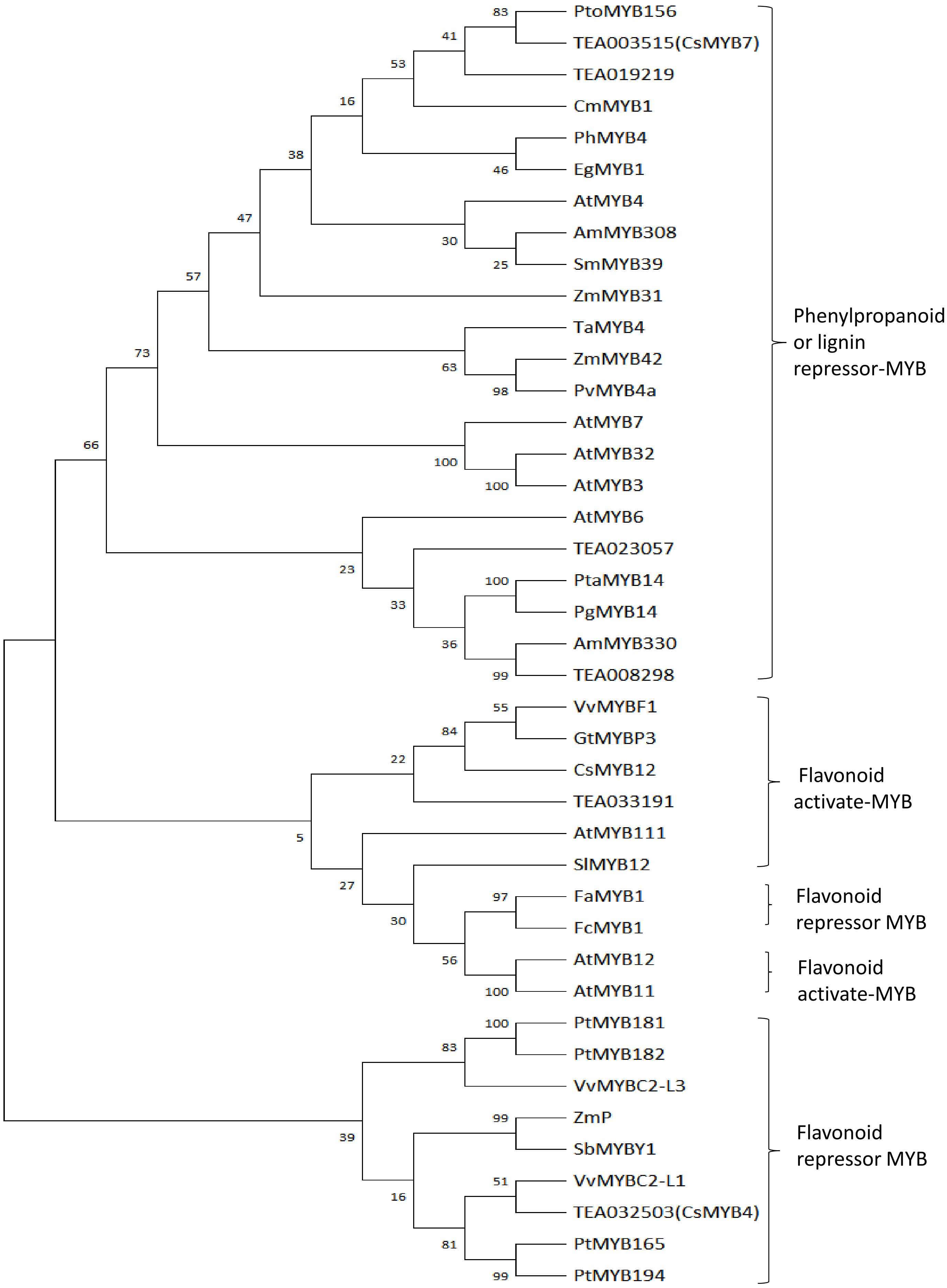


Fig S12. Phylogenetic analysis of CsMYB4,CsMYB7 and MYB homologues from other plants.

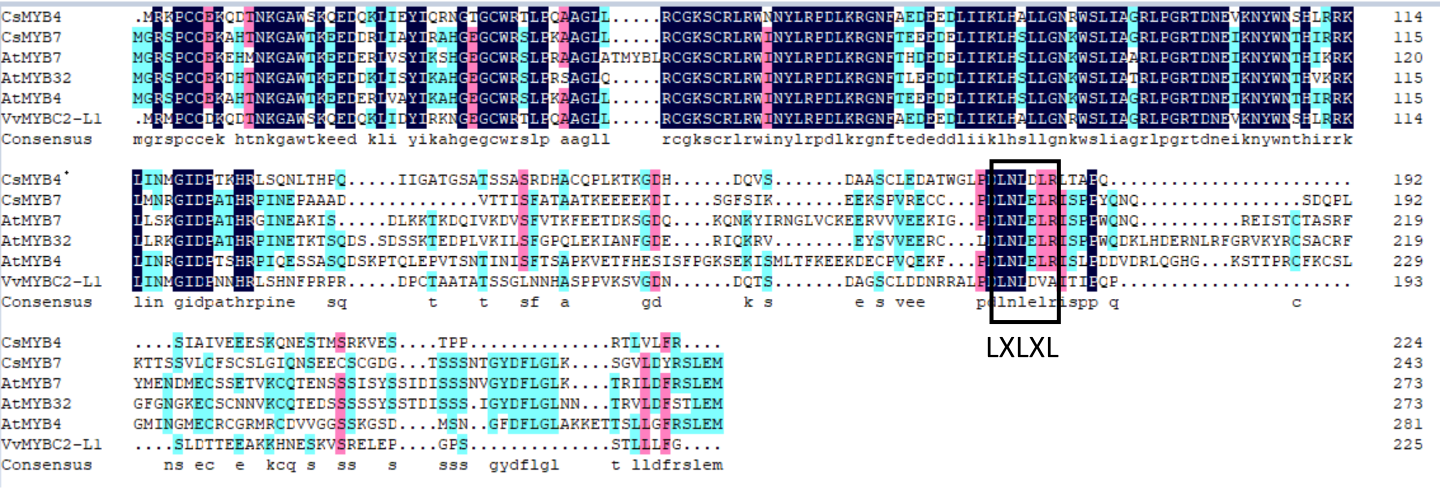


Fig S13. Amino acid sequence alignment of CsMYB4, CsMYB7 and MYB homologues from other plants

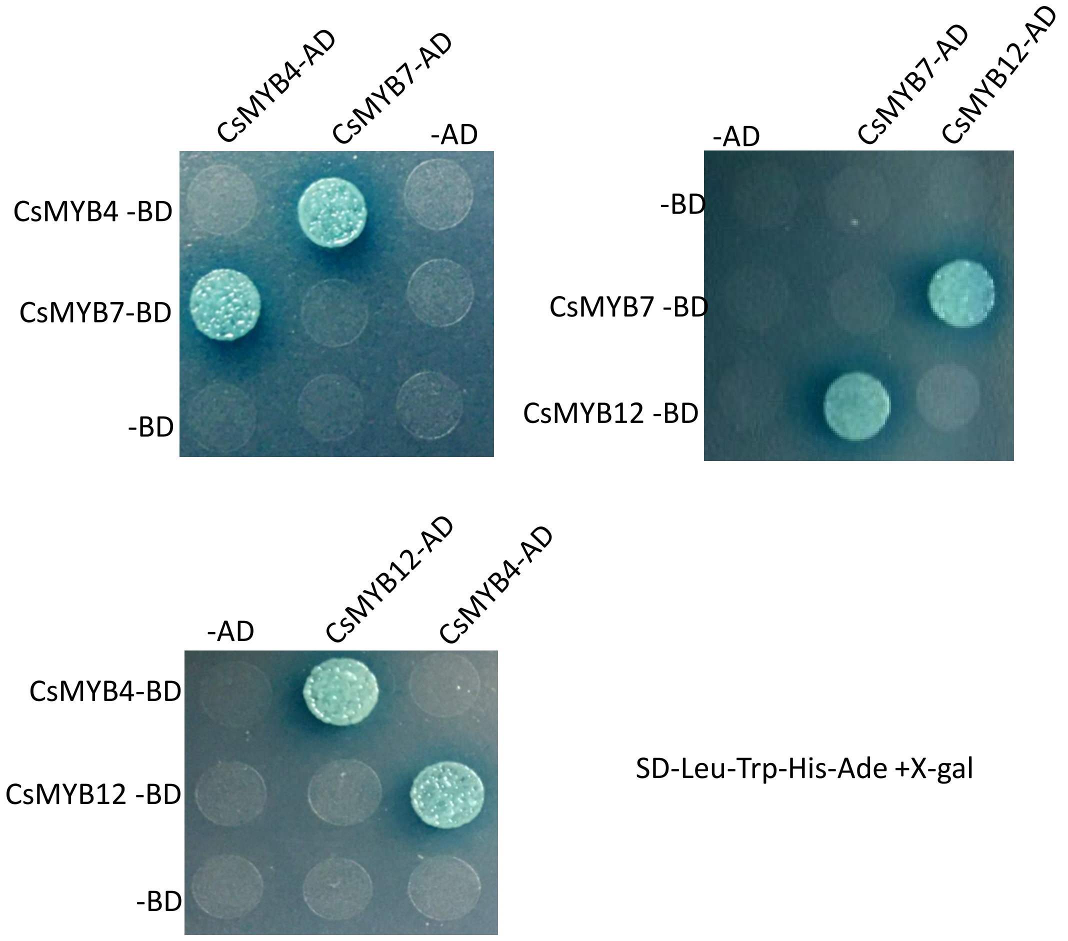


Fig S14. Interactions among CsMYB12, CsMYB4, and CsMYB7 in Y2H assays

(a)

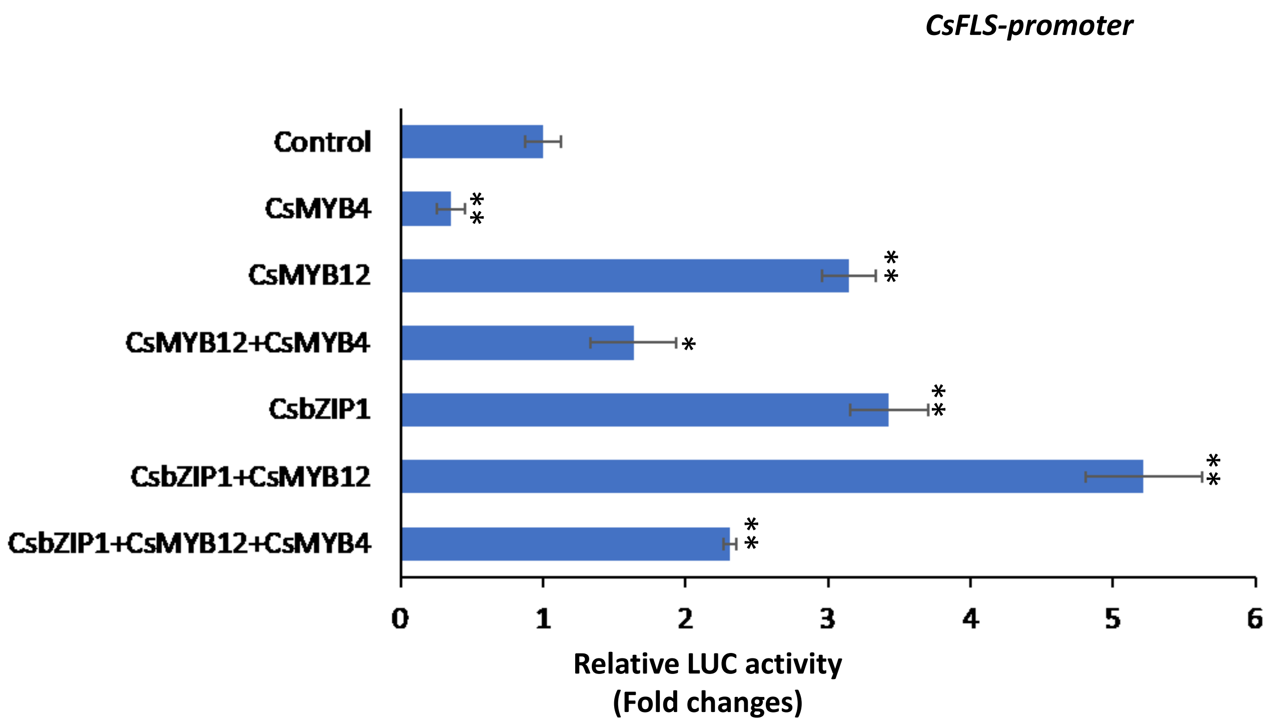


(b)

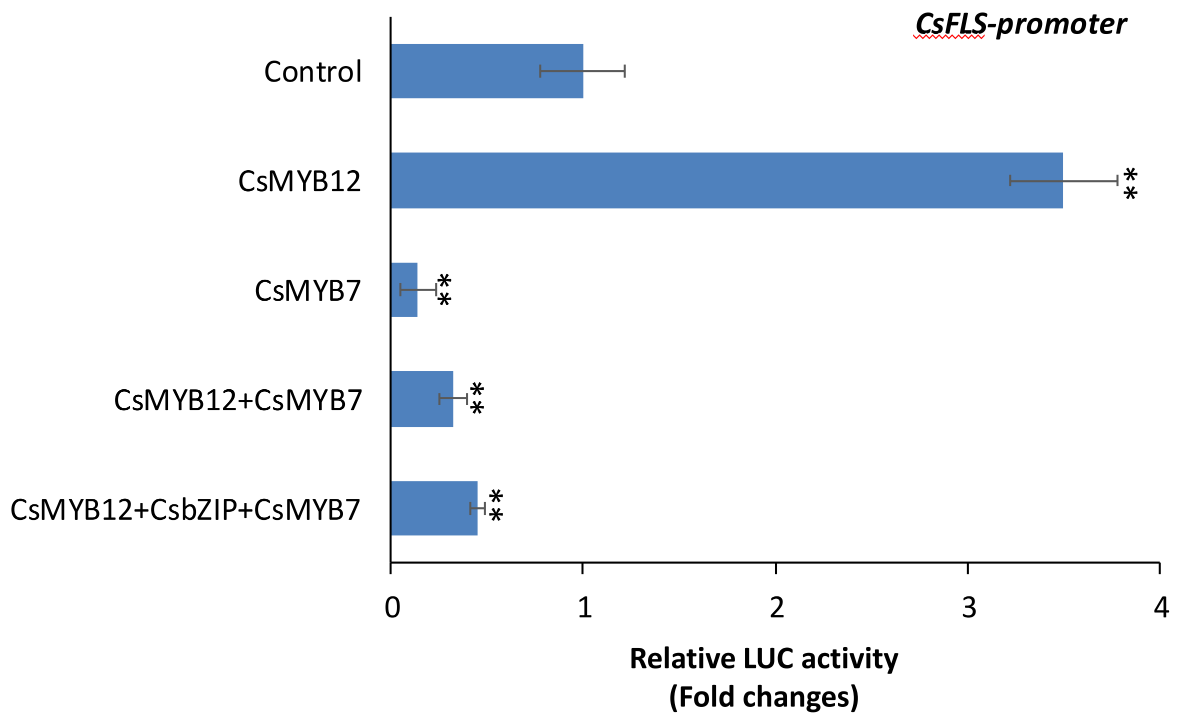


Fig S15. Transactivation assays of CsbZIP1, CsMYB12, CsMYB4 and CsMYB7 individually or in combination on the promoter activity of *CsFLS* in the luciferase reporter assay.

Data were from three independent experiments and expressed as means±S.D. (n = 3). Differences were analyzed, *p < 0.05; **p < 0.01 in student's t-test.

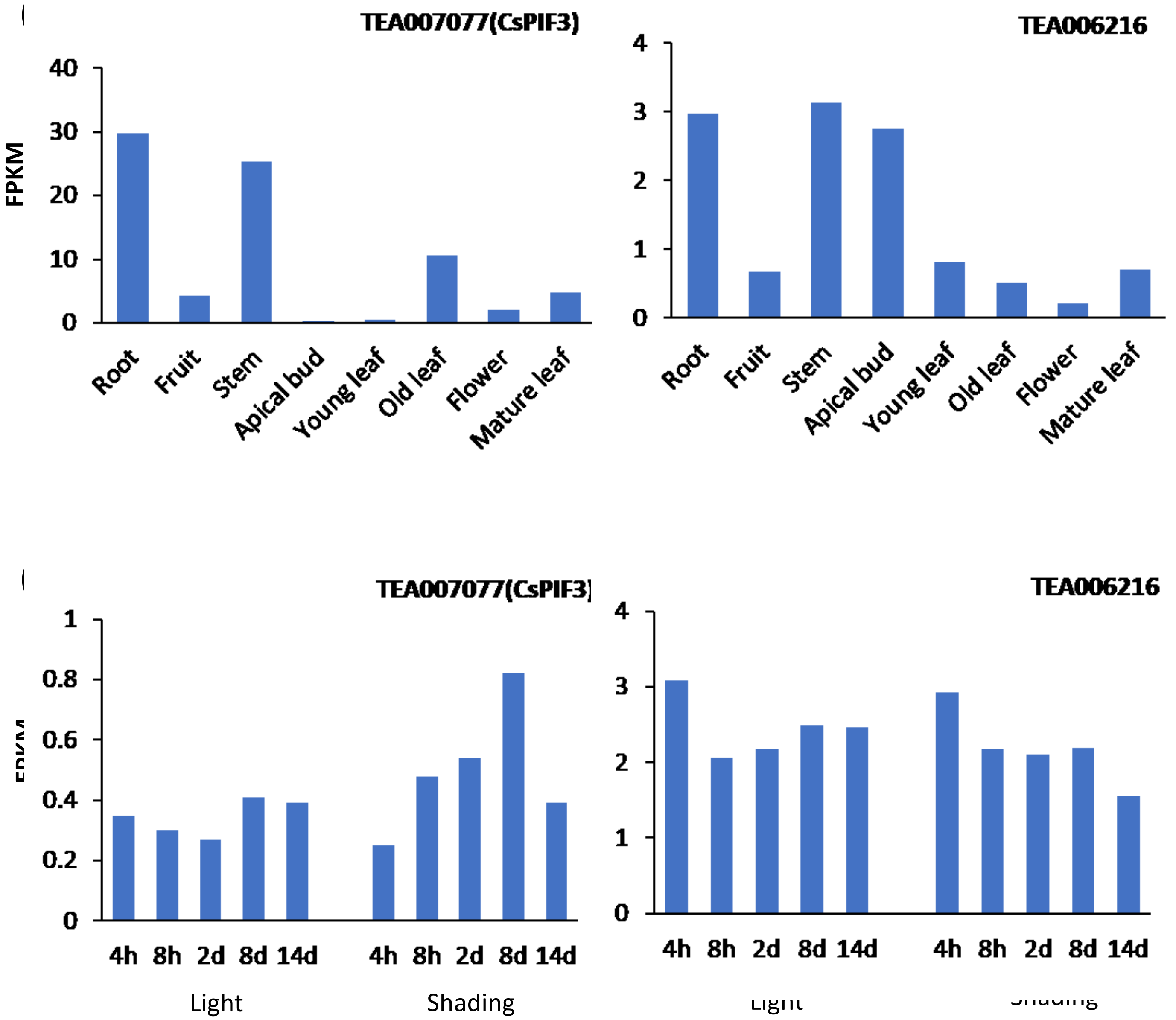


Fig S16. Expression patterns of TEA007077 and TEA006216.

(a) Expression profiles of TEA007077 and TEA006216 in different stages.

(b) Expression patterns of TEA007077 and TEA006216 tea leaf under shading.

```
                                                                        ____
AtPIF1      -------------------------------------------------------STNESR      139
CsPIF3      ESSRARSGQFHDPWLFQEN-QTSIPSLRSKVSGIISNTSCNNKRDAIIVQPNLSRRPNTS      228
AtPIF3      AASSSQYNGFQSHSLYGSDRARDLPSQQT-----------NPDR---FTQTQEPLITSNK      194
                                                                    ..
               APA
            ____________
AtPIF1      PPVRNFMNFSRLRGDF----NNGR--------------GGESGPLLSKAVVRESTQVSPS      181
CsPIF3      SSFMNFPHFSRPSTLVRANLQTIDAITSPGTSNVEPMGGKNKGNDPCSGYFAQS------      282
AtPIF3      PSLVNFSHFLRPATFAKTTNNNLHDTKEKS-------------PQSPPNVFQTR------      235
              . ** :* *         :.                           .

                                                           APB
                                                ________________________
AtPIF8      MSQCVPNCHIDDTPAAATTTVRSTT-------AADIPILDYEVAELTWENGQLGLHGLGP      53
AtPIF1      MHHFVPDFDTDDDYV----NNHNSSLNHLPRKSITTMGEDDDLMELLWQNGQVVVQNQRL      56
CsPIF3      -MPFSEFYGMASGKLESAQQKTTTCLTDL------SHVPHNEFVELVWENGQIMMQGQSS      53
AtPIF3      -MPLFELFRLTKAKLESAQDRN-------------PSPPVDEVVELVWENGQISTQSQSS      46
                        .                           :. ** *:***:  :.
```

Fig S17. Amino acid sequence alignment of CsPIF3 and AtPIF1,AtPIF3 and AtPIF3.

**(a)**

**CACCAAGATCGGCTGAACTCCAGTCTCTCTAACTAACATTCCCCATTTATTCCACCACACATGAGTGATTGTTTA
TCTGCGCGAAGCGCCCACTACTTTTATTTTCAATACTGGCAGCGATCTCGAGGCCCCAATCCCGAGGCGATTTC
ACTAACTCTAAACTCCACGAGAAAAACGTCGGTTGACAGTCACCATGGCATAGTTGTCCAAACGACTTTTTGA
AGAACGTGCACCGTCTGAAAATCTAGGCCCCGAGCCGAGTTGAATTTGCCGTAGCTACCTTACCGACTGGCTT
TTGGCTTCCTCAAGTTACAATAGCCTCAATCAGGGATAAACGGCTATCACGTATGCGTCGACCCTACCGATCAAT
GTTTATACATCCATTCAAACATATACATAAACGAAACGCGTACATACCAAATACGAACTGCAACAAAATATTTAT
CATAGAACGCTTTTTATTTGATAAAACAGGCAATATATTACATAGTTTTCAACGCGATCTACGTTTCAACCTTAC
AACAAGTACACTTATACGGCATCACCCTCATCCTCACTCTTCTCGACCTCAAAAGAAGAAAACGGAAAATGTTT
ACTATGGTAGAATGGCGATGCCGAATCAATGCCCGACGCTCGAAGAGCTTCTGCGGCTTGAGATGCCATGATC
CAACCGTGCCTAAATCCATGATTTTCACATCGGCAAACTTCTTCTACAAGTTTTTCCTTTGTCTTTTTGATTCCAA
CTTTTTGGCCGTCATGAAAGCCTTTCTCATAGGCCCTCCGCTTCGCGTTCTCCAGTGCGAACATACTCGCCGTCT
CACGATCGGCAAGCGCGGAACTGATGAGCCGACTGGTAACCCTTTCTTCCACGATTGGGTTTCTGTGCCTGGC
AGTTTGTGCCTGGGCCGCCTTGGTCTTCCTTAACTCGGTGATTGAATTATTATTATTATTATTATTATTATTAATTC
AAAGTTGTTGAGAGTTACACAAAAGAAAGGCAAAAATAAATAAACACACCGTGTCTTGCTATACTTTAATATA
CCTTACTTTACACCTACCTGAAGATTACCCATAGTTAATGTCCCTATAACTTATTATATGACATTTGATGATGCTAA
TATCATCAAAAGAGAGGCCACACCATCCACTCCCCTCAAACCTGTCTCACCAACTCACATCTAGAAGCAATTAG
GGTCACTTCCCCCCCCTCTCTCTCTCTCTCTCTCTCTCCCTGTATATACATATATATATATATATATATCTATAAAGA
GTCAAAAGGCTATAAAAACCCATCTCAAGTACTCACAGGGGCCGGCCTTGGATTTCCACCATATAACCACTGCA
ACCCAAAGAAAAAGAGAAAGAAGACAAAGAAAAGAAAAGAAAATTGCTAATCAG**

**(b)**

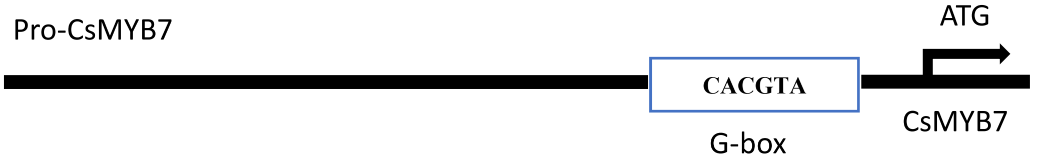


Fig S18. Promoter analysis of CsMYB7.
(a). Promoter sequence of CsMYB7.
(b). The characteristics of cis-elements for binding of *CsMYB7* in the flavonoid gene promoters.

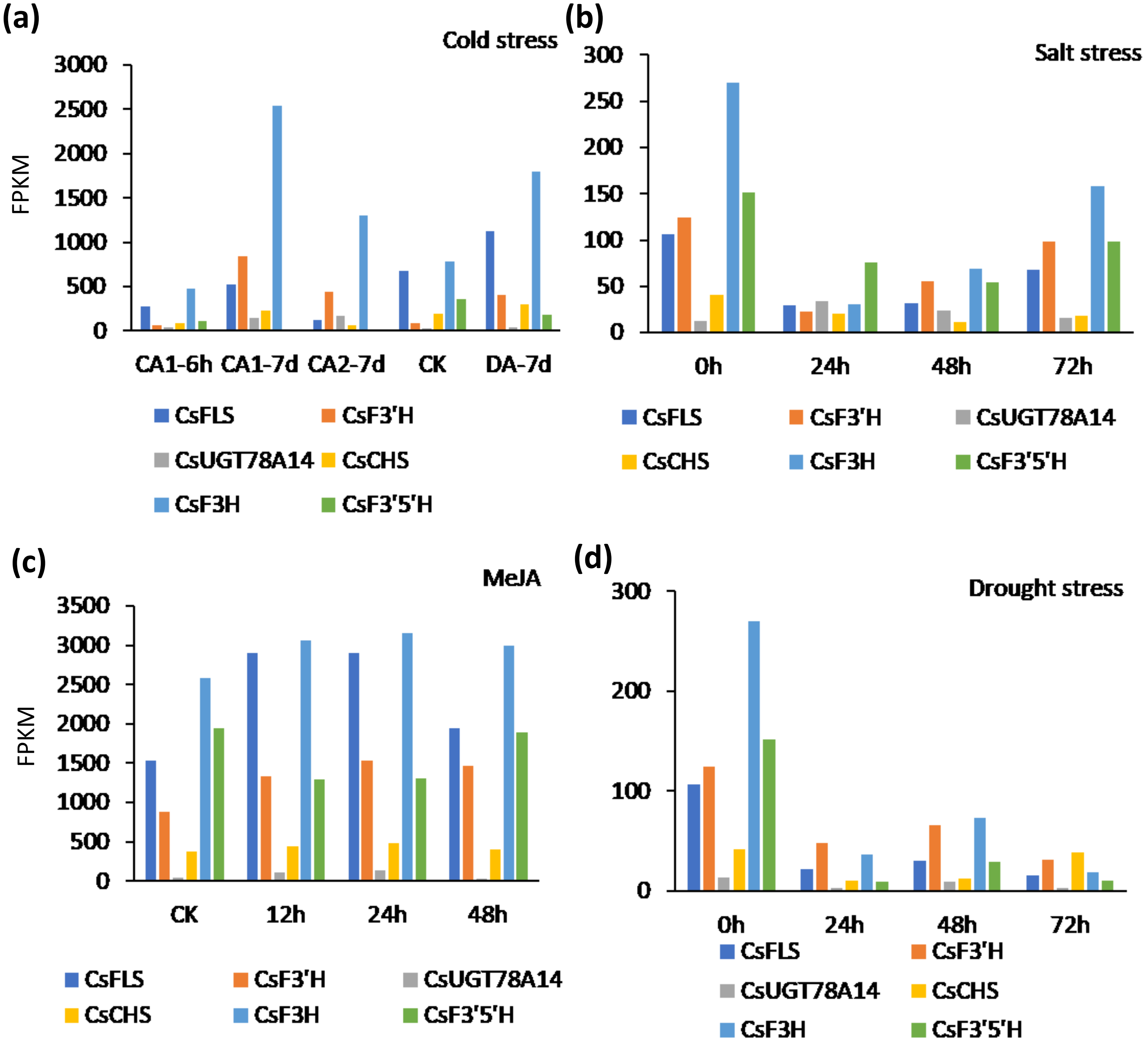


Fig S19. Expression of flavonol structural genes under various abiotic stresses

(a). Expression of *CsFLS, CsF3'H, CsCHS, CsF3H, CsF3'5'H,* and *CsUGT78A14* under cold stress.
(b). Expression of *CsFLS, CsF3'H, CsCHS, CsF3H, CsF3'5'H*, and *CsUGT78A14* under Salt stress.
(c). Expression of *CsFLS, CsF3'H, CsCHS, CsF3H, CsF3'5'H,* and *CsUGT78A14* under MeJA stress.
(d). Expression of *CsFLS, CsF3'H, CsCHS, CsF3H, CsF3'5'H,* and *CsUGT78A14* under PEG treatment.
Transcriptome data from experiments with tea cv. Shuchazao were retrieved from the tea plant information archive (http://tpia.teaplant.org/index.html).

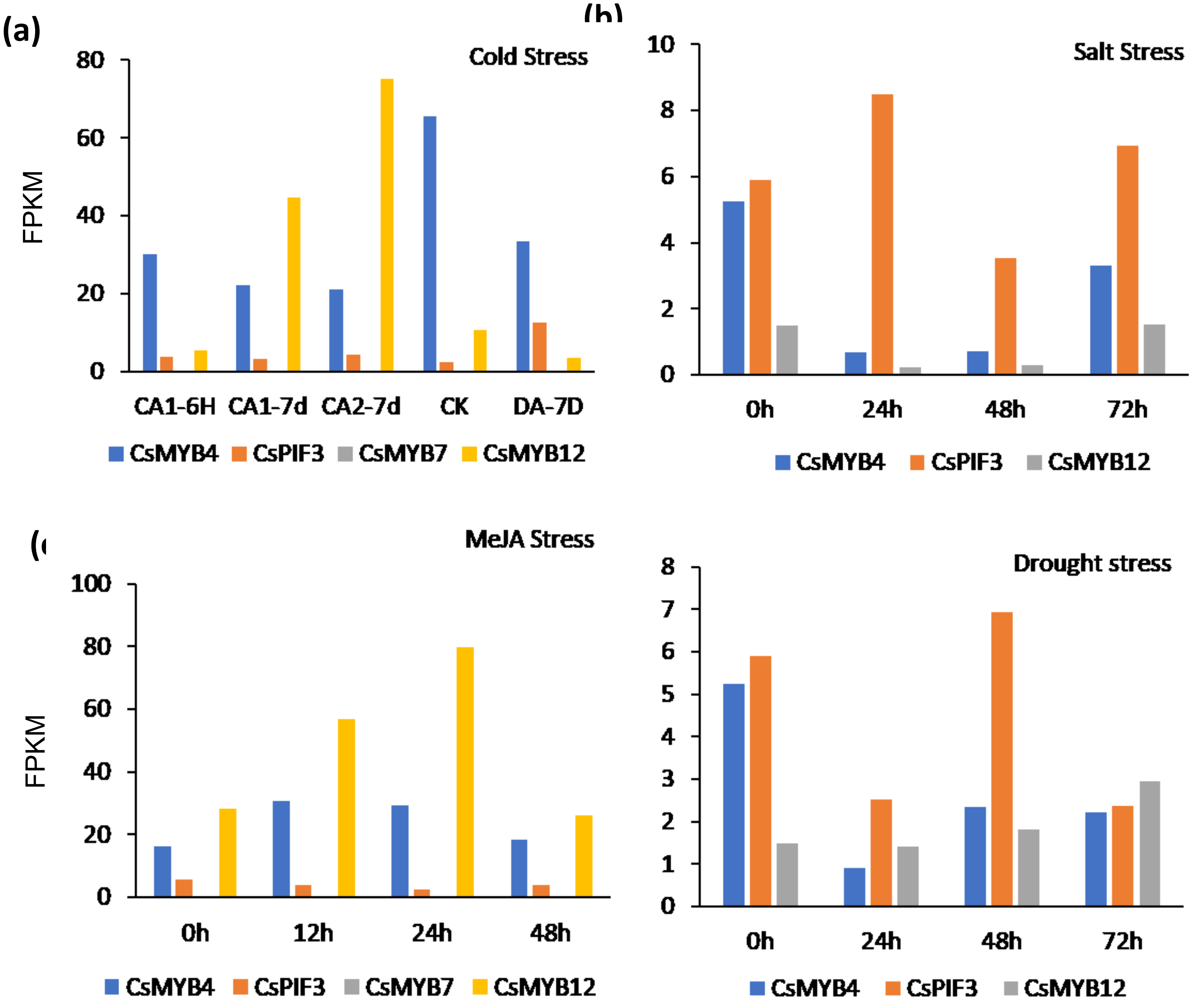


Fig S20. Expression of *CsMYB12,CSMYB4,CsMYB7* and *CsPIF3* in various abiotic stress
(a). Expression *of* *CsMYB12,CSMYB4,CsMYB7* and *CsPIF3* under cold stress.
(b). Expression *of* *CsMYB12, CsMYB4* and *CsPIF3* under Salt stress.
(c). Expression of *CsMYB12, CsMYB4, CsMYB7* and *CsPIF3* under MeJA stress.
(d). Expression *of* *CsMYB12,CsMYB4* and *CsPIF3* under drought stress.
Transcriptome data from experiments with tea cv. Shuchazao were retrieved from the tea plant information archive (http://tpia.teaplant.org/index.html).
